# Proton pump inhibitors attenuate myofibroblast formation associated with thyroid eye disease through the aryl hydrocarbon receptor

**DOI:** 10.1371/journal.pone.0222779

**Published:** 2019-09-19

**Authors:** Christine L. Hammond, Elisa Roztocil, Richard P. Phipps, Steven E. Feldon, Collynn F. Woeller

**Affiliations:** 1 Flaum Eye Institute, School of Medicine and Dentistry, University of Rochester, Rochester, New York, United States of America; 2 Department of Environmental Medicine, School of Medicine and Dentistry, University of Rochester, Rochester, New York, United States of America; Universita degli Studi della Campania Luigi Vanvitelli, ITALY

## Abstract

Thyroid eye disease (TED) can lead to scar formation and tissue remodeling in the orbital space. In severe cases, the scarring process leads to sight-threatening pathophysiology. There is no known effective way to prevent scar formation in TED patients, or to reverse scarring once it occurs. In this study, we show that the proton pump inhibitors (PPIs), esomeprazole and lansoprazole, can prevent transforming growth factor beta (TGFβ)-mediated differentiation of TED orbital fibroblasts to myofibroblasts, a critical step in scar formation. Both PPIs prevent TGFβ-induced increases in alpha-smooth muscle actin (αSMA), calponin, and collagen production and reduce TED orbital fibroblast cell proliferation and migration. Esomeprazole and lansoprazole exert these effects through an aryl hydrocarbon receptor (AHR)-dependent pathway that includes reducing β-catenin/Wnt signaling. We conclude that PPIs are potentially useful therapies for preventing or treating TED by reducing the myofibroblast accumulation that occurs in the disease.

## Introduction

Thyroid eye disease (TED) is a common co-morbidity associated with Graves’ disease and occurs in up to 50% of Graves’ disease patients [1]. Severe TED occurs in approximately 3–6% of those diagnosed and may be sight-threatening [1,2]. The mechanisms behind the initiation and progression of TED are complex and incompletely understood [1–5]. One major contributor to TED is the activation of orbital fibroblasts which alters the composition of the tissue behind the eye [6]. Orbital fibroblasts proliferate and can undergo adipogenesis which results in the accumulation of fat or alternatively, they can differentiate into myofibroblasts leading to scarring, excessive collagen accumulation, and tissue remodeling. These changes, along with the accumulation of hyaluronic acid and the resulting edema that follows, can lead to the protrusion of the eyeball (exophthalmos), which can be disfiguring, painful, and can compress the optic nerve causing vision loss. The eyelids may no longer close properly leading to corneal drying and damage. Orbital scarring affecting the eye muscles can lead to a more severe TED phenotype with double vision (diplopia) and limitation of eye movements. Standard treatments for TED remain corticosteroids and tissue decompression surgery for the severest of patients [2,3]. Teprotumumab and other biological agents are being tested and may offer improved outcomes [7]. Currently, there are no prophylactic treatment options for patients with Graves’ disease to prevent TED. Unfortunately, there are no completely effective pharmaceutical strategies for improving symptoms and reversing the scarring or the fat accumulation for patients with TED [2,5].

Previous work revealed that aryl hydrocarbon receptor (AHR) ligands are beneficial in reducing the orbital fibroblast transition to myofibroblasts after transforming growth factor beta (TGFβ) stimulus. The AHR ligand 2-(1’*H*-indole-3’-carbonyl)-thiaxole-4-carboxylic acid methyl ester (ITE) blocked TGFβ-induced increases in two markers of myofibroblasts, alpha-smooth muscle actin (αSMA) and calponin in TED orbital fibroblasts [8]. ITE also prevented collagen production from TED fibroblasts [8]. However, it was unclear if this beneficial response was dependent upon the AHR itself, as some ligands have independent functions. More recently, we found that the AHR ligand 6-formylindolo[3,2-b]carbazole (FICZ) is also effective against TGFβ-induced myofibroblast formation and collagen production in TED fibroblasts and normal orbital fibroblasts [9]. Importantly, this study demonstrated for the first time that the AHR pathway is critical in preventing myofibroblast formation in TED patient fibroblasts. Further investigation of the mechanism behind FICZ action revealed that the Wnt/β-catenin signaling pathway is critical, and signaling through this pathway was dependent on the AHR [9]. AHR is a ligand-dependent transcription factor with a large binding pocket suggesting there is the opportunity to identify diverse and structurally selective activating ligands [10,11], which could be investigated as potential therapeutics for TED.

In our search for pharmaceutical strategies to reduce orbital scarring and improve outcomes for TED patients, we investigated putative AHR activating pharmaceuticals. One class of drugs that have been reported to have pleiotropic effects linked to the AHR pathway is the proton pump inhibitors (PPIs). These drugs are widely used in the treatment of gastroesophageal reflux disease (GERD) and peptic ulcers [12]. PPIs prevent the proton pump H^+^, K^+^-ATPase from functioning thereby reducing the acidity in the stomach. PPIs are also thought to have anti-inflammatory and anti-fibrotic effects not necessarily due to the actions on H^+^, K^+^-ATPase [13,14]. Omeprazole (Prilosec) is a benzimidazole derivative and an AHR activator [10,15–17]. The PPIs esomeprazole (Nexium) and lansoprazole (Prevacid) have also been indicated as AHR activators and potential ligands [15,18,19]. In this study we investigated the usefulness of esomeprazole and lansoprazole in preventing the production of scar forming TED myofibroblasts; and whether or not these drugs work through the activation of the AHR in TED orbital fibroblasts.

## Materials and methods

### Cell culture

Human primary thyroid eye disease (TED) orbital fibroblasts were isolated from tissue explants collected during decompression surgeries for patients with TED. Informed written consent was obtained from individuals prior to surgery following the guidelines and approval of the University of Rochester Medical School Research Subjects Review Board. Each TED orbital fibroblast strain represents fibroblasts isolated from an individual patient. For each experiment, a minimum of two different strains were used. Fibroblasts were used for experiments at the lowest passage possible, and not exceeding passage 9. Fibroblast strains were cultured in Dulbecco’s modified Eagle’s medium (DMEM) (Invitrogen, Carlsbad, CA) supplemented with 10% fetal bovine serum (FBS) (Hyclone, Logan UT) and antibiotics (Invitrogen, Carlsbad, CA). Orbital fibroblasts were cultured in low serum DMEM (0.1% FBS) for 48 hours prior to experiments. TGFβ (R&D systems, Minneapolis, MN; cat. # 240B or PeproTech, Rocky Hill, NJ; cat. # 100–21) was added to cultures to differentiate orbital fibroblasts to myofibroblasts using 1 ng/mL, unless otherwise noted. The AHR ligand FIZC was purchased from Enzo Life Sciences (Farmingdale, NY; cat. # BML-GR206). The PPIs esomeprazole and lansoprazole were added to cultures either 2 hours or 24 hours prior to TGFβ administration. Esomeprazole and lansoprazole were dissolved in DMSO before administration to the orbital fibroblasts and the untreated control groups included the largest volume of DMSO used as the vehicle for these drugs in experiments. Esomeprazole (cat. # E7906) and lansoprazole (cat. # L8533) were purchased from Sigma-Aldrich (St. Louis, MO).

### Western blot analysis

TED orbital fibroblasts were lysed in a buffer containing: 50 mM Tris-HCl (pH 6.8), 2% SDS and a protease inhibitor cocktail (Sigma-Aldrich, St. Louis, MO). The protein concentration of the cell lysates was determined using the detergent compatible DC protein assay (Bio-Rad, Hercules, CA). Cell lysates containing 5 μg of protein were separated with 10% SDS-PAGE precast gels (Bio-Rad, Hercules, CA), transferred to polyvinylidene difluoride (PVDF) membranes (Millipore, Temecula, CA), and blocked in 5% non-fat dried milk tris-buffered saline solution with 0.1% Tween-20. Antibodies targeting: AHR (rabbit anti-AHR, Cell Signaling Technology, Danvers, MA; cat. # 83200), αSMA (mouse anti- αSMA, Sigma-Aldrich, St. Louis, MO; cat. # A2547), calponin (mouse anti-calponin, Dako, Glostrup, Denmark; cat. # M3556 or Sigma-Aldrich, St. Louis, MO; cat. # C2687), collagen 1a1 (goat anti-collagen, Santa Cruz Biotechnology, Santa Cruz, CA; cat. # sc-8783), CYP1B1 (rabbit anti-CYP1B1, Santa Cruz Biotechnology, Santa Cruz, CA; cat. # sc-32882), phospho-GSK3β (rabbit anti-phosphoGSK3β, Cell Signaling Technology, Danvers, MA; cat. # 9336), GSK3β (rabbit anti-GSK3β, Cell Signaling Technology, Danvers, MA; cat. # 12456), and β-tubulin (rabbit anti-β-tubulin, Cell Signaling Technology, Danvers, MA; cat. # 2146), were diluted according to the manufacturer. Anti-mouse (cat. # 115-035-146), anti-rabbit (cat. # 111-035-144) and anti-goat (cat. # 705-035-147) HRP-conjugated secondary antibodies were all from Jackson Immunoresearch (West Grove, PA). Protein bands were visualized using Immobilon Western chemiluminescent horseradish peroxidase substrate (Millipore, Billerica, MA). Chemiluminescent signals were captured using a VersaDoc Imaging System (Bio-Rad, Hercules, CA). Each panel in a figure represents one Western blot membrane probed multiple times for different proteins. In some cases, a band can be seen beneath the β-tubulin band, which is the αSMA band that was previously detected on that membrane. The densitometry of the bands was determined using ImageLab, version 4 (Bio-Rad, Hercules, CA). The relative expression (RE) of protein bands in each Western blot is reported in figures and compares the intensity of the bands by setting the intensity of the first band on the left (the control) to one. Where bar graphs are included, these depict the mean relative expression for each band in separate experiments and standard error bars are included.

### Immunofluorescence Staining

TED orbital fibroblasts were cultured in 24-well dishes until 90% confluent and then cultured in low serum DMEM (0.1%FBS) for 24 hours. PPIs (25 μM) or vehicle (DMSO) were then added to desired wells for 24 hours before treatment of some wells with 1 ng/mL TGFβ. PPIs were added to the wells every 24 hours for a total of 72 hours post TGFβ treatment. Cells were fixed with 4% paraformaldehyde for 20 minutes, then rinsed in phosphate-buffered saline and permeabilized with 0.3% Triton X-100. TED orbital fibroblasts were blocked with 3% bovine serum albumin, 1% rabbit serum, 3 mM glycine, 0.01% Triton X-100, and 0.1% Tween 20 in phosphate buffered saline for 1 hour. Cells were washed and incubated with Alexa-Fluor 488 conjugated mouse anti-αSMA antibody (Abcam, Cambridge, MA; cat. # ab202295) for 1 hour at room temperature (1:200). Afterwards, cells were washed three times in phosphate buffered saline and counter stained with DAPI nucleic acid binding dye (Invitrogen, Carlsbad, CA). The fluorescence was visualized on a ZOE fluorescent cell imager (Bio-Rad, Hercules, CA) utilizing the same settings for each image in the experiment.

### Collagen slot blot assay

Cell culture supernatant was collected from TED orbital fibroblasts that were treated with vehicle, or treated with PPIs and/or 1 ng/ml TGFβ for 72 hours. A portion (5 μl) of culture supernatant was transferred to a PVDF membrane using a slot blot apparatus (Biometra, Gottingen, GE). The membrane was blocked with 5% nonfat dry milk in phosphate buffered saline with 0.1% Tween-20) and probed with a goat anti-collagen antibody (Santa Cruz Biotechnology, Santa Cruz, CA; cat. # sc-8783) overnight at 4°C. After washing, the blot was incubated with a donkey anti-goat-HRP conjugated antibody (Jackson Immunoresearch, West Grove, PA; cat. # 705-035-147) for 30 minutes. The membrane was developed with chemiluminescent substrate, and imaging was performed on an Azure c500 imager (Azure Biosystems, Dublin, CA). The densitometry of the bands was determined using Image Studio Digits, version 5.2 (LI-COR Biotechnology, Lincoln, NE). The RE was calculated by setting each intensity value of the vehicle treated sample to one.

### Cell viability assay

TED orbital fibroblasts were plated in black 96-well plates (Griener, Sigma-Aldrich) at 5 x 10^3^ cells/well with 200 μl of culture medium. Vehicle (DMSO) or PPIs were added every 24 hours starting with 24 hours before addition of TGFβ (1 ng/ml) for 96 hours to individual wells run in triplicate. Puromycin (5 μg/ml) was used as a positive control for cytotoxicity and was added once. The Alamar blue reagent (Invitrogen, Carlsbad, CA) was added to each well 24 hours after the addition of TGFβ. Fluorescence of the oxidized Alamar reagent was measured after 48 hours (72 hours of incubation with TGFβ and 96 hours with PPIs) (excitation 470 nm, emission 480 nm).

### Cell proliferation assay

Cell proliferation was determined using the bromodeoxyuridine (BrdU) Cell Proliferation Assay kit (Calbiochem, San Diego, CA). TED orbital fibroblasts were seeded in a 96-well plate at a density of 1 x 10^4^ cells/well and cultured in low serum DMEM (0.1% FBS) for 48 hours. Cells were treated in triplicate with vehicle or a PPI for 2 hours, and afterwards TGFβ (1 ng/mL) was added to some of the wells. A BrdU label at a 1:2000 dilution was then added to the cells. The cultures were incubated for 72 hours and the PPIs were added every 24 hours. BrdU incorporation was measured at 450 nm using a Varioskan Flash microplate reader (Thermo Fisher Scientific, Waltham, MA).

### Wound healing (or scratch) assay

TED orbital fibroblasts were seeded in 6 well plates and grown to confluence. Cells were then cultured in low serum DMEM (0.1% FBS) for 48 hours. A wound was made across the wells using a p200 micropipette tip to remove a layer of cells within a straight line. PPIs were added to the wells and cultures were incubated for 2 hours at 37°C. The wound area was imaged at 8 recorded locations as a baseline measurement using bright-field microscopy. TGFβ (5 ng/mL) was added to wells and cultures were returned to incubate at 37°C. PPIs were added to the wells every 24 hours. At 72 hours and 120 hours the same 8 locations were imaged of the wound area. The area of each wound was measured using ImageJ software (NIH, Bethesda, MD) and the data from the 8 sampled areas averaged for each treatment. Wound areas at 72 hours and 120 hours were normalized to the baseline area of the wound.

### RNA extraction and quantitative PCR

Total cell RNA was extracted using Qiazol lysis reagent (Qiagen, Valencia, CA) and isolated with an RNeasy Mini Kit (Qiagen, Valencia, CA). Total RNA concentrations were determined with a NanoDrop 1000 spectrophotometer (Thermo Scientific, Wilmington, DE). cDNA was generated using the iScript reverse transcription kit (Bio-Rad, Hercules, CA) and gene expression was quantified via real-time PCR with gene specific primers, a universal SsoFast Evergreen PCR master mix (Bio-Rad, Hercules, CA) and the BioRad CFX Connect Real-Time System (Bio-Rad, Hercules, CA). Gene specific primers are as follows: CYP1B1 (CYP1B1 fwd 5’-CTATCACTGACATCTTCGGCG-3’) and (CYP1B1 rev 5’CATACAAGGCAGACGGTCC-3’), 18S rRNA (18S fwd 5’-TGAGAAACGGCTACCACATC-3’) and (18S rev 5’-ACTACGAGCTTTTTAACTGC-3’).

### Gene expression knockdown using siRNA

*AHR* siRNA 1 and 2 (siRNA ID numbers: s1198 and s1200) and a non-specific, negative control siRNA (negative control #1) were purchased from Ambion’s Silencer Select pre-designed siRNA library (Ambion, Grand Island, NY). Cells were grown in 6-well plates to approximately 70% confluence and treated with the siRNAs mixed with Lipofectamine 2000 (Invitrogen, Carlsbad, CA) in OptiMEM I (Invitrogen, Carlsbad, CA) at a final concentration of 100 nM for 24 hours. Cells were incubated in low serum DMEM (0.1% FBS) for a further 24 hours prior to PPI treatment. TGFβ (1 ng/mL) was added 24 hours after the PPI where indicated.

### Luciferase reporter assays

HEK293FT cells were transfected with plasmid DNA using Lipofectamine 2000 (Invitrogen, Carlsbad, CA). These plasmids include: M50 Super 8× TopFlash-luciferase plasmid (β-catenin/Wnt reporter) (Addgene, Cambridge, MA plasmid 12456), the pCMV-human *AHR* cDNA plasmid and the pCMV-empty plasmid. After 24 hours, cells were seeded in 96-well plates and treated with PPIs for 6 hours before the addition of 500 ng/mL of Wnt3a (R&D Systems, Minneapolis, MN; cat. # 5036-WN). Cells were further incubated for 18 hours and then cells were lysed directly in plates using Steady-Glo reagent (Thermo Fisher Scientific, Waltham, MA). Firefly luciferase readings were measured on a Varioskan Flash luminescent plate reader (Thermo Fisher Scientific, Waltham, MA) and are reported as relative light units.

### Statistical analysis

All results are reported as mean +/- standard error of the mean. One-way and two-way ANOVAs with Tukey’s Post Hoc analysis were performed using the GraphPad Prism software, version 7 (GraphPad, San Diego, CA). Statistical significance is denoted by p values of p < 0.05 (* or #); p < 0.01 (** or ##); p < 0.001 (*** or ###) or p < 0.0001 (**** or ####).

## Results

### PPIs inhibit the TGFβ-mediated differentiation of TED orbital fibroblasts to myofibroblasts

**Fig 1:**
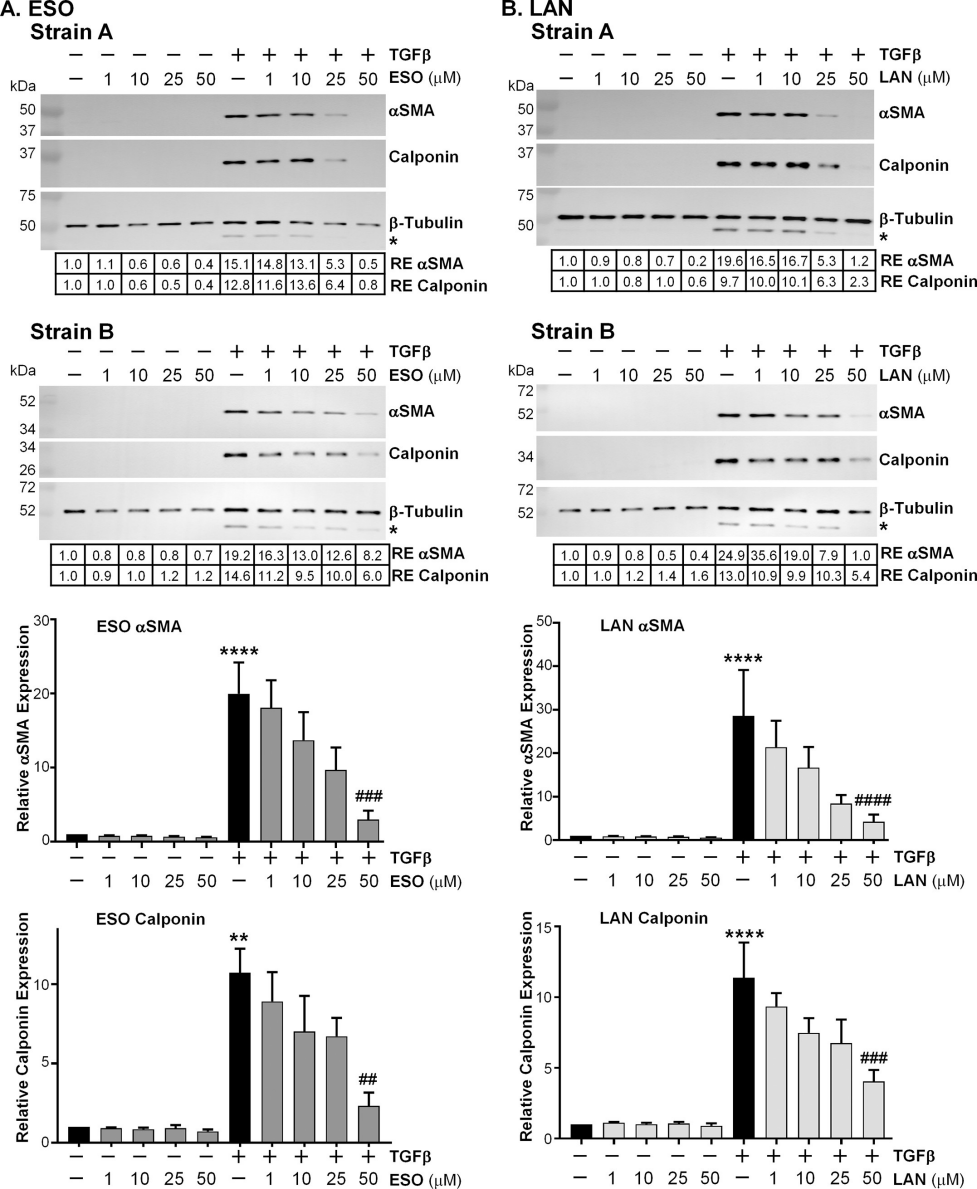
PPIs prevent TGFβ-induced myofibroblast marker expression. A) PPIs were added to TED orbital fibroblasts daily for 4 days. TGFβ (1 ng/mL) was administered after the first 24h of PPI exposure. Cell lysates were collected after the fourth day and protein levels of αSMA and calponin were measured by Western blot. A) Two different representative strains of TED orbital fibroblasts were treated with esomeprazole +/- TGFβ. Esomeprazole led to a dose-dependent prevention in TGFβ-mediated αSMA and calponin increase. RE = relative expression of protein bands compared with first band on the left. * The faint band beneath the β-tubulin band is αSMA that was previously detected. B) The same two representative TED orbital fibroblast strains were treated with lansoprazole +/- TGFβ. Lansoprazole also led to a dose-dependent prevention in TGFβ-mediated αSMA and calponin increase. RE = relative expression of protein bands compared with first band on the left. * The faint band beneath the β-tubulin band is αSMA that was previously detected. Bar graphs depicting the dose-dependent effects of the PPIs on αSMA and calponin expression due to TGFβ are presented beneath the Western blots. N = 4–6 different TED orbital fibroblast strains. ** = p < 0.01; **** = p < 0.0001; significantly different from vehicle treated control. ## = p < 0.01; ### = p < 0.001; #### = p < 0.0001; significantly different from TGFβ alone. Statistics were measured by one-way ANOVA and Tukey’s post hoc analysis.

Orbital fibroblasts can be induced to differentiate into myofibroblasts through TGFβ signaling, a critical step in scarring. To initiate myofibroblast formation, we treated TED orbital fibroblasts with 1 ng/mL TGFβ for 72 hours and measured the protein expression of αSMA and calponin, two hallmarks of myofibroblast differentiation [8, 9, 20, 21]. Two PPIs, esomeprazole and lansoprazole, were tested using dose-response experiments to determine the effectiveness in reducing TGFβ-mediated myofibroblast marker expression. The PPIs were added 24 hours before 1 ng/mL TGFβ. The PPIs were added daily throughout the experiment. Cell lysates were collected on the third day after TGFβ was added and analyzed for myofibroblast marker protein expression using Western blotting. This experiment was repeated in six strains of TED orbital fibroblasts explanted from orbital tissue removed from different human patients. Representative Western blots from two different strains are shown in Fig 1. TGFβ led to a marked increase in αSMA and calponin in both Strains A and B (compare lanes 1 and 6 in Fig 1). The TED orbital fibroblast response to TGFβ is dramatic but does vary between strains and experiments (for example, there is a 15–25 fold increase in the αSMA band intensity due to TGFβ shown in Strains A and B). This variation is also apparent when averaging the relative expression from multiple strains in bar graphs; however, a clear dose-dependent response was observed from both PPIs in reducing αSMA and calponin expression due to TGFβ.

Esomeprazole began to reduce αSMA and calponin at the 1 μM dose. In Strain A, the 25 μM esomeprazole and TGFβ band intensity (Fig 1A, Strain A, lane 9), was one-third of the intensity of the band generated from TGFβ alone for αSMA and one-half of the calponin band (Fig 1A, Strain A, lane 6). At the 50 μM dose of esomeprazole and TGFβ, both αSMA and calponin bands were below the intensity of the control band (Fig 1A, Strain A, lane 10 versus lane 1). For Strain B, the αSMA response to TGFβ was higher than in Strain A and an effect from esomeprazole on TGFβ was again seen starting at the 1 μM dose (Fig 1A, Strain B, lane 7), with the largest effects seen at the 50 uM dose, which was less than one-half the expression of TGFβ alone (Fig 1A, Strain B, lane 10 vs. lane 6). The calponin response to TGFβ was also a little higher in Strain B than in Strain A; esomeprazole was again able to reduce expression of calponin with 50 μM esomeprazole to less than one-half of TGFβ alone (Fig 1A, Strain B, lane 10 vs. lane 6).

Lansoprazole was also effective in reducing TGFβ-mediated αSMA and calponin upregulation starting at the 1 μM dose (Fig 1B). In Fig 1B, TGFβ increased both αSMA and calponin, with the TGFβ-induced αSMA and calponin bands intensity higher in Strain B than in Strain A, consistent with the results seen in Fig 1A. In Strain A, lansoprazole decreased the TGFβ-mediated αSMA band intensity starting at the 1 μM dose, and by the 25 μM dose the αSMA band is one-fourth as strong as TGFβ alone. The 50 μM dose of lansoprazole nearly prevents the TGFβ-mediated increase in αSMA. Calponin was also reduced by lansoprazole in Strain A, the intensity of the 25 μM lansoprazole and TGFβ band was two-thirds of the TGFβ band (Fig 1B, Strain A, lane 9 versus lane 6) and the intensity of the 50 μM lansoprazole and TGFβ band was one-third of the TGFβ band (Fig 1B, Strain A, lane 10 versus lane 6). Strain B showed a similar response. The TGFβ-mediated increase in αSMA was partially prevented by 10 μM lansoprazole and the band from 25 μM lansoprazole and TGFβ was one-fourth as strong as TGFβ alone. The TGFβ-mediated increase in αSMA was fully prevented by 50 μM lansoprazole (Fig 1B, Strain B, lane 10). The TGFβ-mediated increase in calponin was reduced by more than half with 50 μM lansoprazole. These results indicate that the PPIs, esomeprazole and lansoprazole, are effective in reducing myofibroblast marker upregulation initiated by TGFβ in TED orbital fibroblasts.

**Fig 2:**
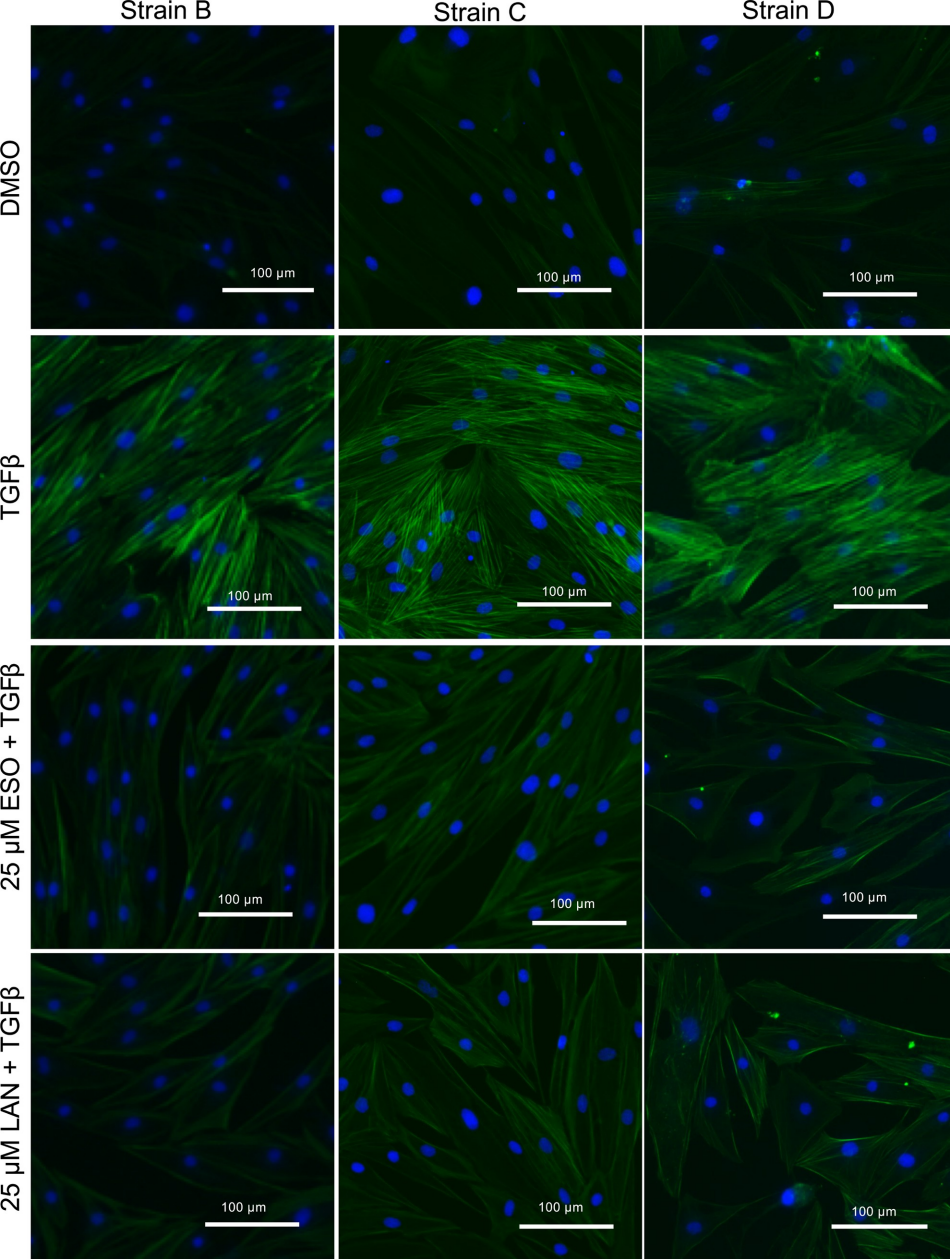
PPIs prevent the TGFβ-induced myofibroblast marker αSMA expression. PPIs (25 μM) were added to TED orbital fibroblasts 24h before 1ng/mL TGFβ and then every 24h for the next 72h. TGFβ-induced αSMA expression was measured with fluorescent imaging. Green = αSMA; Blue = DAPI staining of nuclei. Three representative strains are shown. The bar on the images represents 100 μm.

Next, immunofluorescence was performed in TED orbital fibroblasts to provide further evidence that both esomeprazole and lansoprazole are effective in preventing TGFβ-mediated αSMA accumulation. TED orbital fibroblasts were treated with PPIs (25 μM) for 24 hours before TGFβ was added to the cultures. The PPIs were also added with the TGFβ, and then daily for 2 more days. Afterwards, the fibroblasts were fixed and stained with anti-αSMA antibody conjugated to AlexaFluor 488, which stains the αSMA filaments green (Fig 2). The fibroblasts were also counter-stained with DAPI, which stains the fibroblast nuclei blue. Fibroblasts treated with DMSO showed blue nuclei staining and no green αSMA staining (Fig 2, top row). In marked contrast, fibroblasts treated with TGFβ show bright green αSMA staining among the blue nuclei (Fig 2, second row). Esomeprazole and lansoprazole at the 25 μM dose showed a dramatic decrease in the green staining (Fig 2, third and fourth rows). Although some background staining is present, the bright green actin filaments were dramatically reduced when either esomeprazole or lansoprazole were added to TGFβ-treated TED orbital fibroblasts.

**Fig 3:**
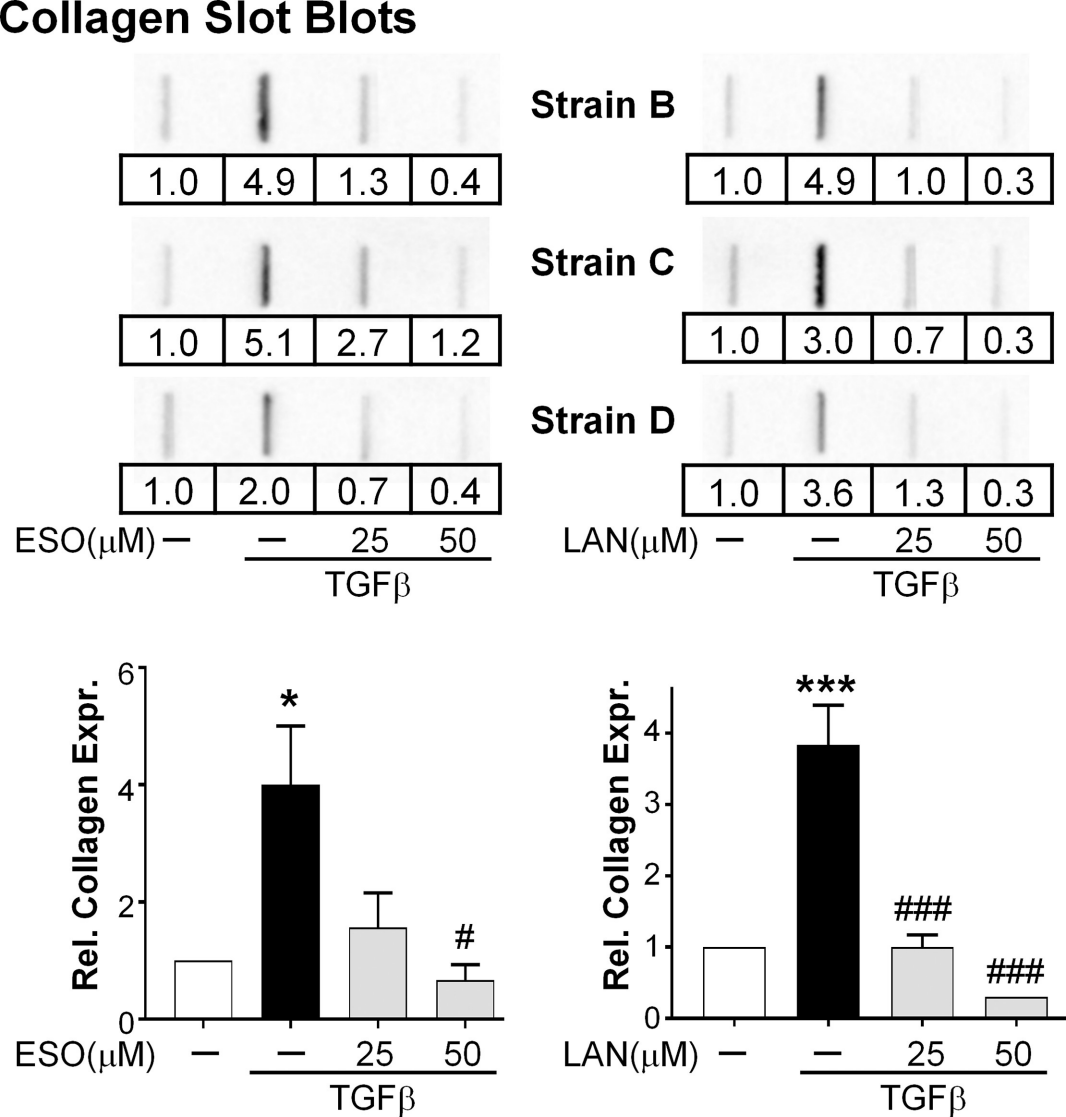
PPIs attenuate TGFβ-induced collagen production. PPIs were added to TED orbital fibroblasts 24h before 1 ng/mL TGFβ and then every 24h for the next 72h. Supernatants were collected at the end of the 72h and measured for collagen content using collagen slot blots. Data from three representative strains are shown. RE = relative expression; determined by setting each vehicle treated sample in the group of four bands to one. Graphs represent data from images of Strains B-D. N = 3. * = p < 0.05; *** = p < 0.001; significantly different from vehicle treated control. # = p < 0.05; ### = p < 0.001; significantly different from TGFβ alone. Statistics were measured by one-way ANOVA and Tukey’s post hoc analysis.

Another aspect of myofibroblasts is that they produce ample amounts of collagen, increasing the volume of the ECM [20]. Esomeprazole and lansoprazole were tested for their ability to attenuate TGFβ-mediated collagen α1 type 1 production. TED orbital fibroblasts were treated with 25 μM or 50 μM esomeprazole or lansoprazole for 24 hours before TGFβ was added. The PPIs were added with TGFβ and then again daily for the next two days. After 3 days in culture, the presence of collagen α1 type 1 in the cell culture supernatant was measured using a collagen slot blot. Equal volumes (5 uL) of supernatant were loaded onto the slot blot and then collagen was detected with an anti-collagen α1 type 1 antibody (Fig 3). Three representative slot blots from different TED fibroblast strains are shown for each PPI. TGFβ led to a significant increase in the amount of collagen α1 type 1 present in the cell culture media. Both esomeprazole and lansoprazole prevented this increase effectively, with the 25 μM dose added to TGFβ returning the levels to near the control levels. The 50 μM dose of PPIs added to TGFβ reduced the collagen α1 type 1 amounts in the supernatant to below the basal levels produced by the controls.

**Fig 4:**
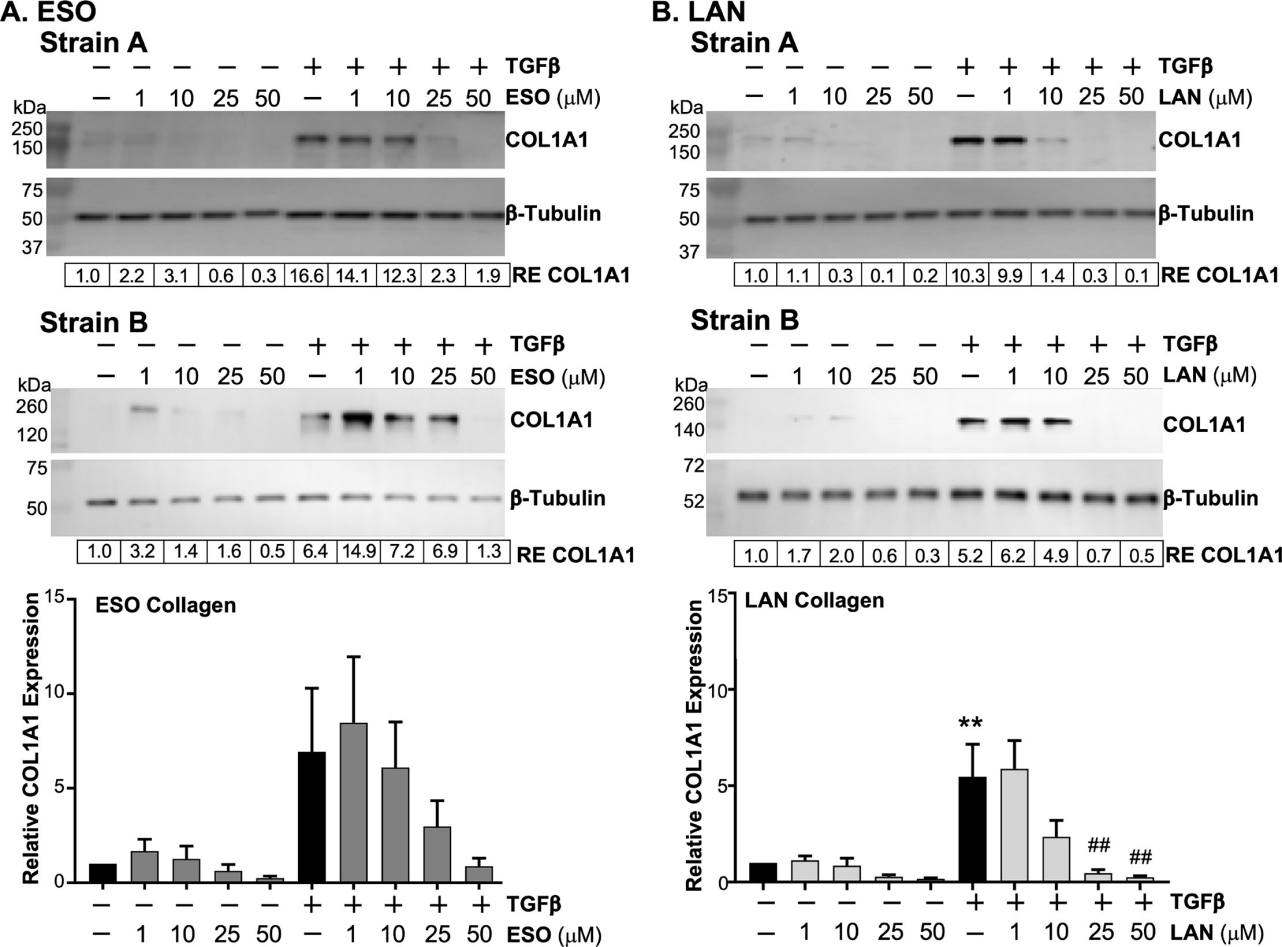
PPIs attenuate cell associated collagen induced by TGFβ. PPIs were added to TED orbital fibroblasts 24h before 1ng/mL TGFβ and then every 24h for the next 72h. Cell lysates were collected and measured for collagen 1A1 using Western blotting. Western blots from two representative TED orbital fibroblast strains are shown. A) Orbital fibroblasts treated with esomeprazole (ESO). B) Orbital fibroblasts treated with lansoprazole (LAN). RE = relative expression of protein bands compared with first band on the left. Bar graphs of the mean relative expression for each band depict a dose-dependent reduction in both basal and TGF-induced collagen levels and are shown beneath the Western blots. N = 4 different TED orbital fibroblast strains. ** = p < 0.01; significantly different from vehicle treated control. ## = p < 0.01; significantly different from TGFβ alone. Statistics were measured by one-way ANOVA and Tukey’s post hoc analysis.

Not all collagen produced by the fibroblasts is released into the supernatant as some may stay associated with the cells themselves. Therefore, cell associated collagen α1 type 1 levels were also measured in the cell lysates of TED orbital fibroblasts using Western blotting to determine the effects of the PPIs (Fig 4). TED orbital fibroblasts were treated with 1 μM to 50 μM esomeprazole or lansoprazole 24 hours prior to the addition of TGFβ. The PPIs were added again with the TGFβ and then daily for 2 more days. On the third day, cell lysates were collected and collagen α1 type 1 was measured using anti-collagen α1 type 1 antibody (COL1A1). The COL1A1 antibody from Santa Cruz measures the collagen α1 type 1 precursor between 140–210 kDa, and we detect a band using this antibody just above the 140–150 kDa markers. Esomeprazole reduced basal levels of collagen α1 type 1 in both strains (Fig 4A). Esomeprazole was effective in preventing TGFβ-mediated collagen α1 type 1 expression most notably in both strains at the 25 μM and 50 μM doses, which corresponds with the doses that were effective in preventing myofibroblast marker expression (compare Fig 4A with Fig 1A). In Strain A, the intensity of the COL1A1 band was near control levels with 25 μM esomeprazole and TGFβ as well as 50 μM esomeprazole and TGFβ. The 1 μM and 10 μM doses of esomeprazole also partially prevented TGFβ-induced COL1A1 production. In Strain B, TGFβ-mediated COL1A1 levels were near background levels at the 50 μM dose. Similar results were obtained in two additional TED fibroblast strains. The mean relative expression of each band in the four separate experiments is depicted in the bar graphs beneath the representative blots (Fig 4).

For lansoprazole, the noticeable decrease in TGFβ-mediated collagen α1 type 1 production occurred at the 10 μM dose in Strain A and 25 μM dose in Strain B, which brought the intensity of the COL1A1 band back to near or below background levels (Fig 4B). The COL1A1bands for 25 μM lansoprazole and TGFβ in Strain A and 50 μM lansoprazole and TGFβ in both strains were nearly absent. Basal levels of COL1A1 were also decreased by lansoprazole. Both esomeprazole and lansoprazole prevent TGFβ-mediated collagen production (shown in Fig 3 and Fig 4) and myofibroblast marker expression (Fig 1 and Fig 2) in TED orbital fibroblasts and taken together they provide strong evidence that these PPIs can prevent TGFβ-mediated myofibroblast formation.

### PPIs do not significantly reduce TED orbital fibroblast viability

**Fig 5:**
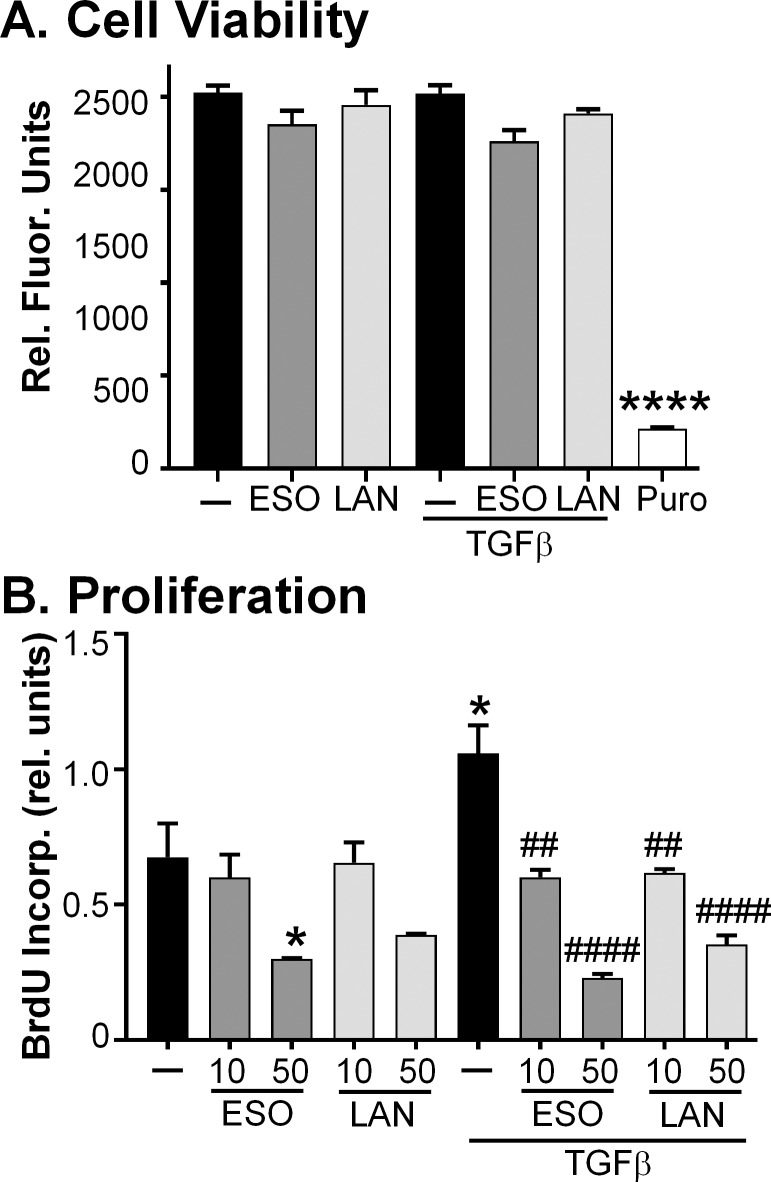
PPIs do not reduce TED orbital fibroblast cell viability; however, they do reduce fibroblast proliferation. A) PPIs (ESO = esomeprazole; LAN = lansoprazole; 50 μM dose) were added to cell cultures every 24h for 96h. TGFβ (1 ng/mL) was added 24h after the first PPI dose. Cell viability in TED orbital fibroblasts was measured using the Alamar Blue assay. PURO = Puromycin, a positive control for cell death. N = 3 different TED orbital fibroblast strains. **** p < 0.0001; significantly different from all other treatments measured with a one-way ANOVA and Tukey’s post hoc analysis. B) Cells were treated with PPIs (ESO = esomeprazole; LAN = lansoprazole; 10 and 50 μM doses) for 2h before some cultures received the addition of 1 ng/mL TGFβ. The PPIs were then added every 24h afterwards for a total of 72h after TGFβ administration. Cellular proliferation was measured with BrdU incorporation. Basal and TGFβ-induced fibroblast proliferation was attenuated by the PPIs. N = 3 different TED orbital fibroblast strains. * p < 0.05; significantly different from vehicle treated control. ## p < 0.01; #### p < 0.0001; significantly different from TGFβ treatment alone. Statistics were measured with a one-way ANOVA and Tukey’s post hoc analysis.

The doses of PPIs administered daily to the fibroblasts did not affect cell viability as measured by the Alamar Blue assay (Fig 5). PPIs were administered to TED orbital fibroblasts daily at the 50 μM dose for 4 days with and without TGFβ treatment for the last 3 days as described above. Alamar blue was added to the cultures for the last 2 days of treatment. A dose of puromycin known to cause a severe loss in cell viability was included as a positive control (PURO in Fig 5A). Puromycin led to a drastic decrease in the fluorescent signal of Alamar blue compared with control (300 versus 2000, respectively; Fig 5A). Conversely, the esomeprazole and lansoprazole treatments with and without TGFβ were all near 2000, suggesting no great loss in cell viability at the highest doses of PPIs used in these experiments. We also tested the 1, 10, and 25 μM concentrations of PPIs in the Alamar blue assay and observed no loss in cell viability.

### PPIs inhibit TGFβ-mediated proliferation and migration in TED orbital fibroblasts

Another characteristic of TED is aberrant cellular proliferation which contributes to the expansion of orbital tissue as a whole and also to the myofibroblast population. TGFβ can activate orbital fibroblasts and lead to proliferation, and esomeprazole and lansoprazole were tested for their ability to prevent this proliferation using the BrdU assay (Fig 5B). TED orbital fibroblasts were pretreated with the PPIs, and some wells were also treated with TGFβ and then the BrdU was added to the cells. The PPIs were added daily to the cultures for 3 days, and then the BrdU incorporation was measured using a plate reader. Both esomeprazole and lansoprazole at 50 μM doses decreased basal levels of cell proliferation by approximately one-half and the decrease caused by 50 μM esomeprazole reached statistical significance. Adding TGFβ to the TED orbital fibroblasts nearly doubled the amount of proliferation when compared with control. The PPIs added with TGFβ decreased the amount of proliferation back to basal levels. Esomeprazole and lansoprazole at the 10 μM dose decreased TGFβ-induced proliferation by almost one-half. TGFβ-induced proliferation was reduced by about one-fifth with 50 μM esomeprazole and one-third with 50 μM lansoprazole.

**Fig 6:**
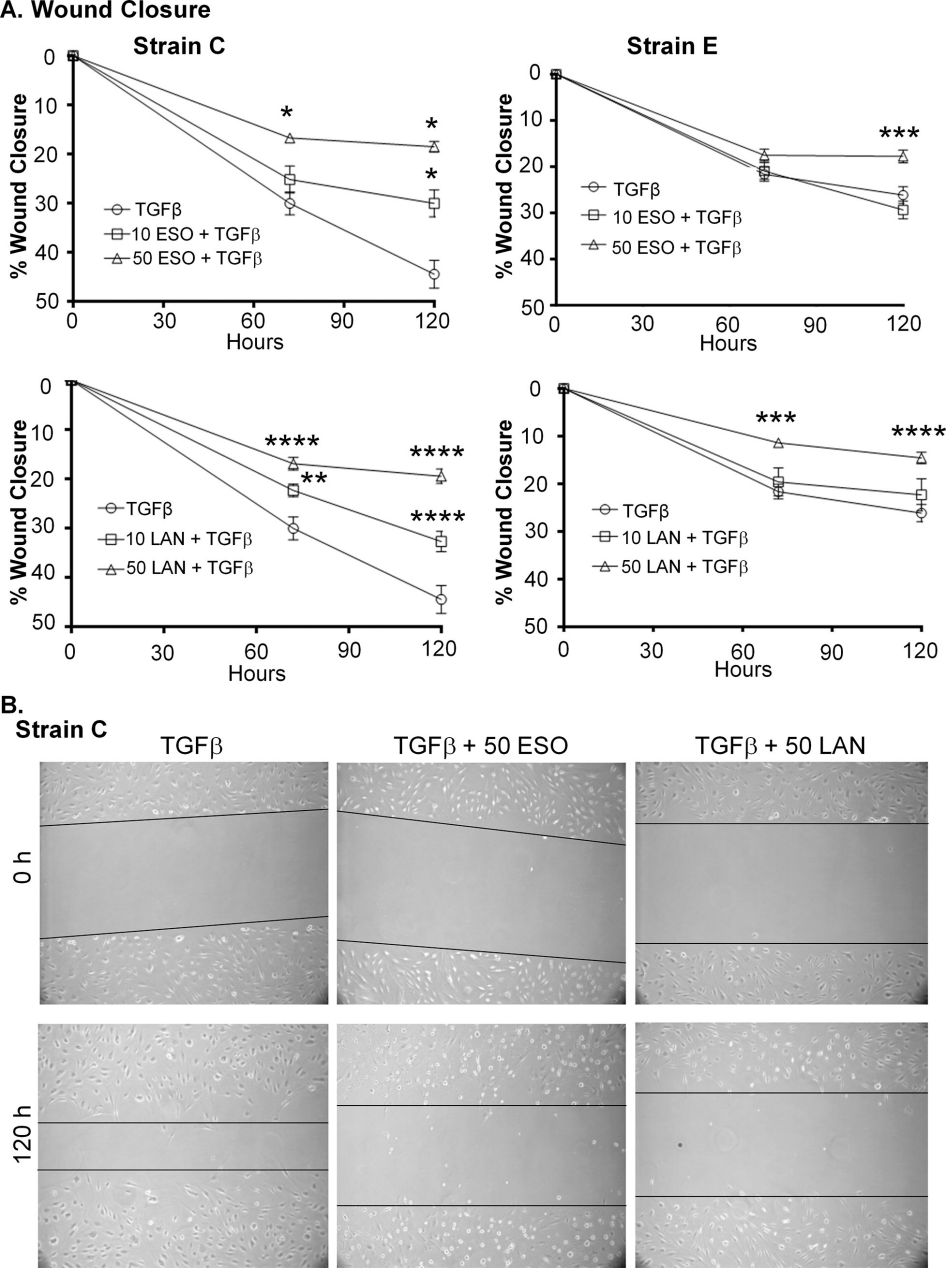
PPIs attenuate TED orbital fibroblast proliferation and migration in a wound healing assay. Wounds were scratched across confluent TED orbital fibroblasts and bright-field pictures were taken at specific locations across the wound, the area of the wound was determined and set at 0% wound closure. Cells were treated with PPIs for 2h before TGFβ (5 ng/mL) was added. The PPIs were added every 24h afterwards until 120h. Additional pictures were taken at 72 and 120h of the same specific locations imaged directly after the scratch. The wound area was determined using Image J software and is reported as relative to the wound area at time zero. A) Line graphs for two representative strains are shown. ESO = esomeprazole and LAN = lansoprazole. * p < 0.05; ** p < 0.01; *** p < 0.001, **** p < 0.0001; significantly different from TGFβ treatment alone. N = 8 technical replicates. Statistics were measured with a two-way ANOVA and Tukey’s post hoc analysis. B) Representative bright-field pictures of the wounds across the TED orbital fibroblasts in Strain C, demonstrating the prevention of fibroblast migration after TGFβ treatment by esomeprazole (ESO) and lansoprazole (LAN).

To further investigate esomeprazole and lansoprazole’s effects on proliferation and migration, a wound healing (or scratch) assay was performed (Fig 6). A single scratch was generated in confluent wells of TED orbital fibroblasts (see Fig 6B, top row). Fibroblasts were then treated with TGFβ with and without 10 or 50 μM doses of esomeprazole or lansoprazole. The PPIs were added 2 hours prior to TGFβ and then daily afterwards for 5 days. The area of the scratch was then measured and graphed as a percentage of the initial scratch area (Fig 6A). In Strain C, TGFβ caused approximately 30% of the scratch to fill in after 3 days and nearly 50% of the scratch was closed after 5 days. Both esomeprazole and lansoprazole had dose-dependent effects on preventing the TGFβ scratch closure. After 3 days, 50 μM esomeprazole prevented approximately one-half the wound closure due to TGFβ, at about 15% and this stayed nearly stable at the fifth day. The lesser dose of esomeprazole also prevented about one-third the wound closure at 5 days. Lansoprazole appeared to be more effective then esomeprazole, at 3 days the wound closure in the 10 μM lansoprazole and TGFβ treated fibroblasts was just over 20% and at the 50 μM lansoprazole and TGFβ dose was approximately 15% compared with the 30% of TGFβ alone. For representative images of Strain C see Fig 6B.

In Strain E, TGFβ was not as effective in promoting wound closure as in Strain C, only reaching about 25% closed. Nevertheless, the 50 μM doses of esomeprazole and lansoprazole were able to reduce TGFβ-mediated wound closure in Strain E. The 50 μM esomeprazole and TGFβ treatment was approximately 15% closed at 5 days; whereas, the 50 μM lansoprazole and TGFβ treatment was nearly 10% closed at both 3 and 5 days. Taken together, the data presented in Fig 5B showing that PPIs reduce TGFβ-mediated proliferation and the data in Fig 6 showing that the PPIs reduce TGFβ-mediated proliferation and migration in the wound healing assay provide strong evidence that the PPIs would be beneficial in preventing the proliferation of fibroblasts during TED.

### PPIs are AHR activators in TED orbital fibroblasts

**Fig 7:**
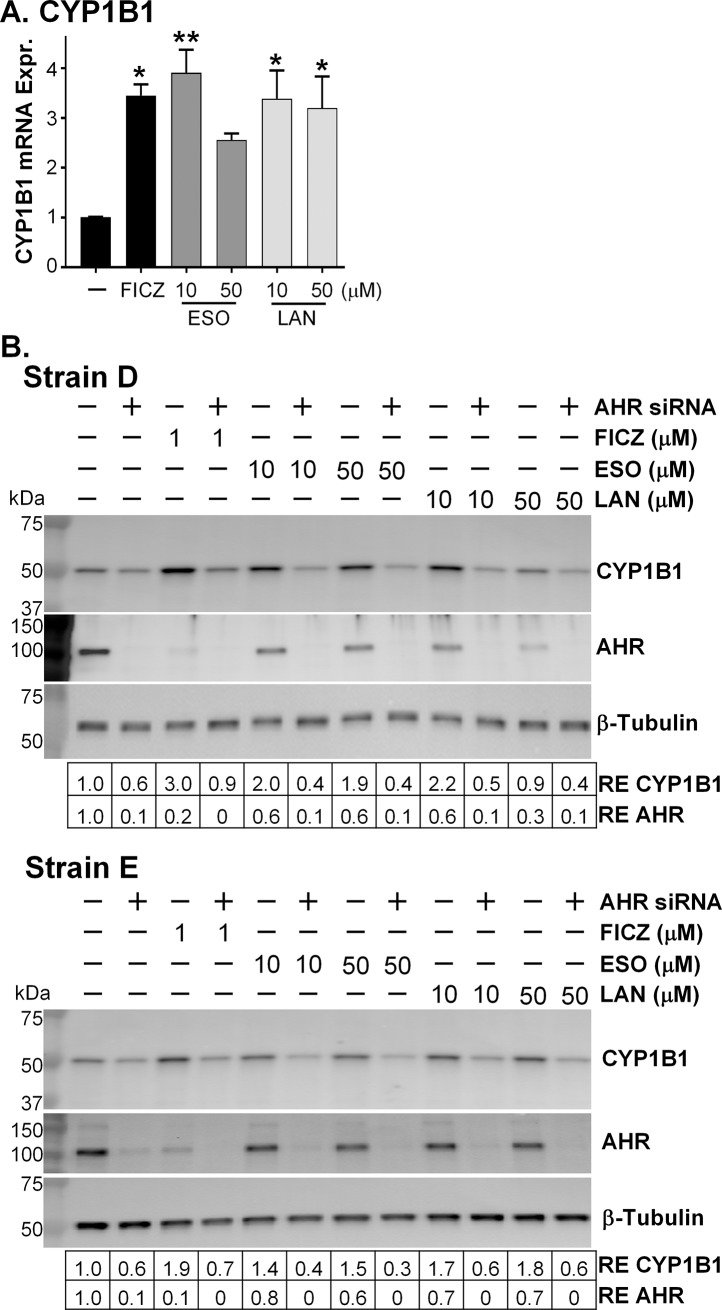
PPIs are AHR activators. A) *CYP1B1* mRNA expression after 6h PPI treatment. Cells were treated with an AHR ligand positive control (FICZ) or proton pump inhibitors for 24h before RNA was isolated from cell lysates and analyzed for *CYP1B1* mRNA expression using qPCR. ESO = esomeprazole; LAN = lansoprazole. N = 3 different TED orbital fibroblast strains. * p < 0.05; ** p < 0.01; significantly different from vehicle treatment alone. Statistics were measured with a one-way ANOVA and Tukey’s post hoc analysis. B) After reduction of *AHR* expression with *AHR* specific siRNA, FICZ, esomeprazole or lansoprazole were added for 24h. Cell lysates were analyzed for CYP1B1, AHR, and β-tubulin protein expression using Western blotting. Two representative TED orbital fibroblast strains are shown, Strain D and Strain E. RE = relative expression of protein bands compared with first band on the left.

PPIs have been proposed to be AHR activators, yet the effect of compounds on AHR can differ depending on the tissue or cell line [10,11]. Therefore, we sought to provide evidence that the PPIs were working as AHR activators in TED orbital fibroblasts and that this was the mechanism behind the beneficial effects of PPIs in preventing TGFβ-induced myofibroblast formation. We measured the expression of *CYP1B1*, a key gene that is upregulated by AHR signaling, in TED orbital fibroblasts after 6 hours of 10 or 50 μM PPI treatment using qPCR. Both esomeprazole and lansoprazole increased *CYP1B1* mRNA expression above the control cells in a similar fashion as FICZ, a compound known to be an AHR activator in TED orbital fibroblasts and used as a positive control (Fig 7A) (9). FICZ increases *CYP1B1* expression 3.5-fold higher than control. Esomeprazole at 10 μM and lansoprazole at 10 and 50 μM were also near this same level of induction. The higher dose of esomeprazole also increased *CYP1B1* expression about 2.5-fold over control.

CYP1B1 protein expression was also measured to examine the effects of the PPIs and to determine if these effects are dependent on the presence of the AHR. TED orbital fibroblasts were treated with control or *AHR* specific siRNA to reduce *AHR* expression. Two different *AHR* siRNAs that target unique regions of the *AHR* gene were tested with equal success [9]. The Western blots in Fig 7B show how efficient the *AHR* specific siRNA was in reducing AHR protein levels, every odd lane contains the control siRNA samples and every even lane the *AHR* siRNA samples. The relative expression of AHR in most of the even lanes is near zero. The third lane in the Western blots show the increase in CYP1B1 protein expression with the known AHR activator FICZ. The increase is AHR dependent because the increase in CYP1B1 expression due to FICZ is lost when the *AHR* siRNA was used (compare lanes 3 and 4, Fig 7B). Esomeprazole and lansoprazole increased CYP1B1 expression in the control siRNA lanes (lanes 5, 7, 9, and 11, Fig 7B) and remained at control expression levels in the *AHR* siRNA lanes (lanes 6, 8, 10, and 12, Fig 7B) demonstrating the requirement for *AHR* to increase CYP1B1. Note also in lane 3 that FICZ decreases AHR expression, indicating that it is an AHR ligand, as previous reports have indicated that AHR protein is degraded after ligand binding and transcriptional activation [22]. Both esomeprazole and lansoprazole also reduce AHR protein expression compared with control (compare lanes 5, 7, 9, and 11 with lane 1, Fig 7B) providing further evidence that the PPIs are AHR activators.

### PPIs prevent the differentiation of TED orbital fibroblasts to myofibroblasts in an AHR-dependent manner in part by blocking the phosphorylation of GSK3β

**Fig 8:**
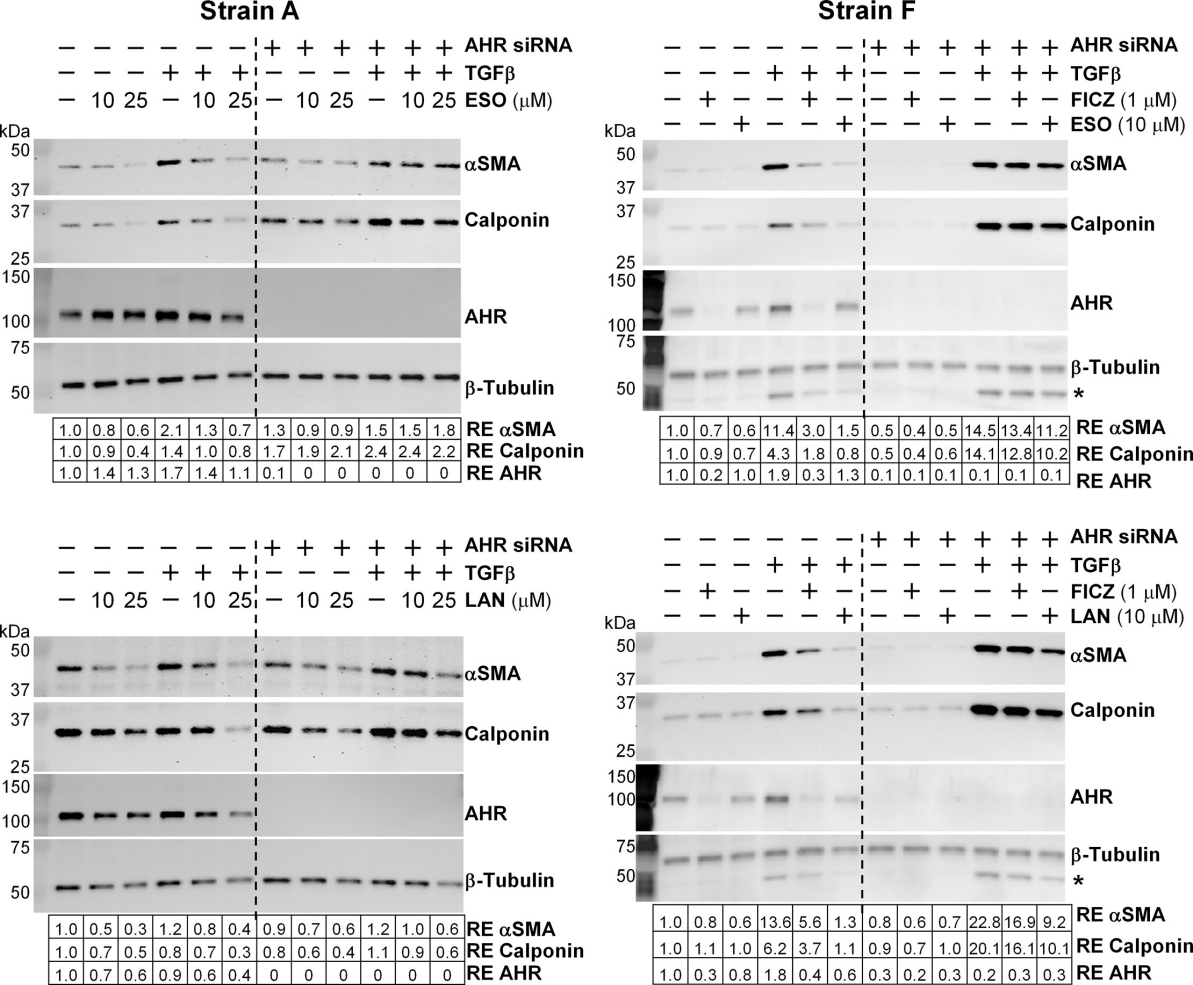
PPIs prevent TGFβ-induced myofibroblast marker expression in an AHR-dependent manner. After reduction of AHR expression with *AHR* specific siRNA, FICZ or a proton pump inhibitor (ESO = esomeprazole; LAN = lansoprazole) was added every 24h for 96h. After the first 24h, 1 ng/mL TGFβ was added. Cell lysates were collected at the end of 96h and analyzed for αSMA, calponin, AHR, and β-tubulin expression using Western blotting. Two representative strains of different TED orbital fibroblasts are shown, Strain A was used in the left two panels and Strain F was used in the right two panels. RE = relative expression of protein bands compared with first band on the left. * The band beneath the β-tubulin band is αSMA that was previously detected on this membrane.

The data presented above provide evidence that esomeprazole and lansoprazole are AHR activators in TED orbital fibroblasts. To determine if the mechanism that the PPIs use to prevent myofibroblast formation involves AHR signaling, we again used *AHR* specific siRNA technique to deplete TED orbital fibroblasts of the AHR. We then tested whether the PPIs continued to inhibit the TGFβ-mediated increase of αSMA and calponin. Four representative Western blots are shown of two different TED orbital fibroblast strains, the top two were treated with esomeprazole and the bottom two were treated with lansoprazole (Fig 8). The lines through the center of the Western blot panels indicate that the left six bands are control siRNA samples and the right six bands are *AHR* specific siRNA samples. All four Western blots demonstrate that AHR protein was successfully decreased by the *AHR* siRNA; the relative expression of AHR is at or near zero. The AHR protein band in most instances was also decreased by the PPIs and FICZ, providing additional evidence that the PPIs are AHR activators like FICZ. The left 6 lanes in the Western blots confirm what was reported in Fig 1, that esomeprazole and lansoprazole inhibit the TGFβ-mediated increase in both αSMA and calponin (for Strain A, compare lanes 5 and 6 with lane 4 and Strain F compare lane 6 with lane 4, Fig 8). In the right 6 lanes where AHR is depleted through *AHR* specific siRNA, esomeprazole and lansoprazole no longer inhibit the increase in αSMA and calponin in the TGFβ treated samples (compare lane 12 to lane 6 in each panel; Fig 8). These results indicate that esomeprazole and lansoprazole are reducing TGFβ-mediated myofibroblast formation in an AHR-dependent manner.

**Fig 9:**
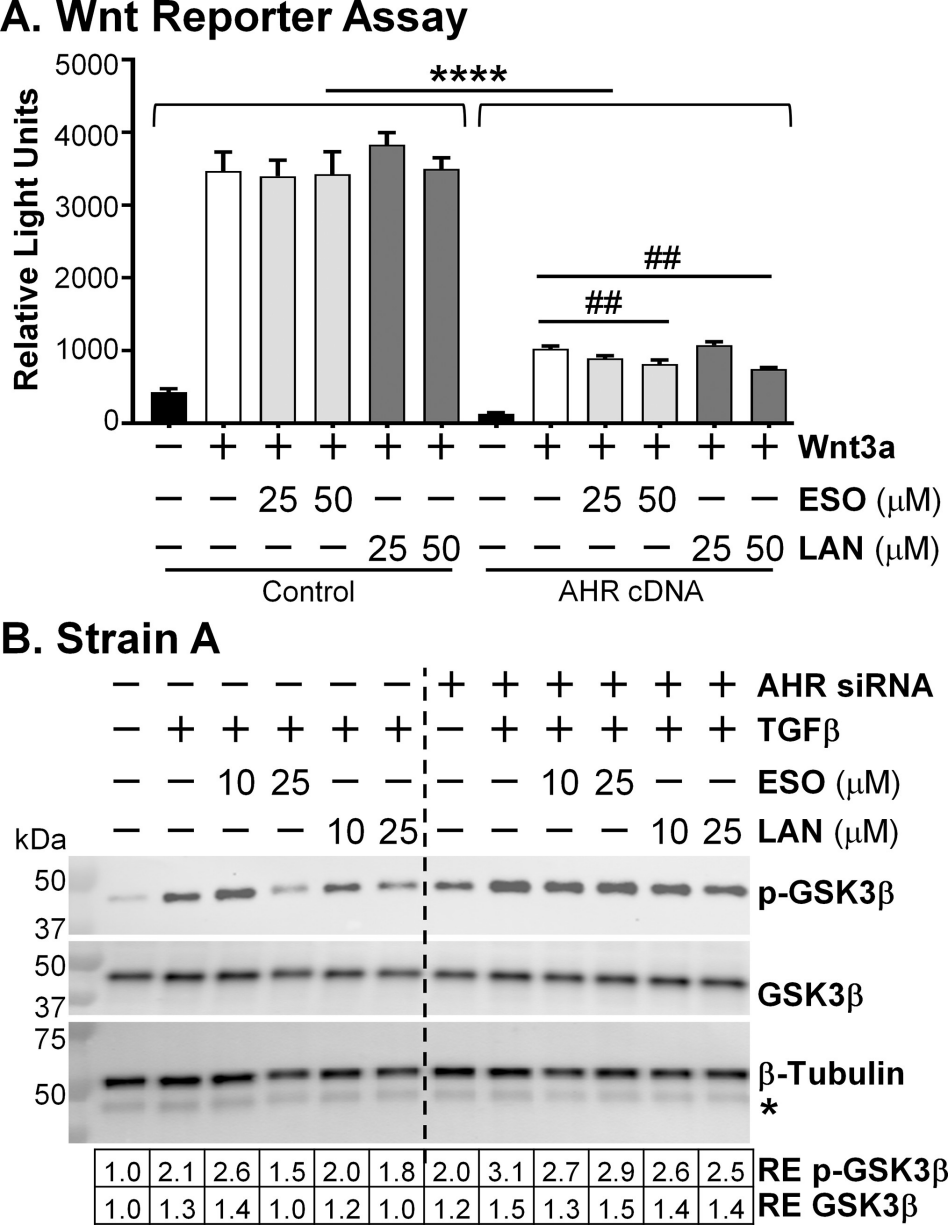
PPIs reduce Wnt signaling in an AHR-dependent manner. A) Wnt reporter assay. HEK293FT cells were used in a reporter assay to monitor Wnt signaling in the presence or absence of exogenous AHR expression. This experiment was repeated three times and a representative experiment is shown. N = 3–6 technical replicates. **** p < 0.0001; each control plasmid bar is significantly higher than its respective AHR cDNA plasmid bar. ## p < 0.01; 50 μM PPIs are significantly lower than Wnt treatment alone in the AHR cDNA plasmid group. Statistics were measured with a one-way ANOVA and Tukey’s post hoc analysis. B) Western blot for phospho-GSK3β. After reduction of AHR expression with *AHR* specific siRNA, esomeprazole or lansoprazole was added every 24h for 96h. After the first 24h, 1 ng/mL TGFβ was added for the remainder of the 72h. Cell lysates were collected and analyzed for phosphorylated GSK3β, total GSK3β, and β-tubulin expression using Western blotting. RE = relative expression of protein bands compared with first band on the left. * The band beneath the β-tubulin band is GSK3β that was previously detected on this membrane.

With our previous work with the AHR ligand FICZ in TED orbital fibroblasts, we showed that FICZ blocked the β-catenin/Wnt pathway in an AHR-dependent manner by reducing the phosphorylation of GSK3β and disrupting TGFβ-mediated myofibroblast formation [9]. We investigated whether esomeprazole and lansoprazole were also blocking the β-catenin/Wnt pathway using a Wnt reporter assay (Fig 9A). HEK293FT cells were used in this reporter system. These cells are deficient in AHR expression, are readily transfected, grow quickly, are easily analyzed for reporter activation and our lab has previously used this system to investigate two additional AHR activators, FICZ and ITE [9]. HEK293FT cells were transfected with the Top-flash luciferase reporter construct and either the pCMV empty control plasmid or the pCMV-human AHR cDNA plasmid. The transfected cells were treated with the PPIs for 6 hours and then Wnt was added and the cells further cultured for 18 hours before the luciferase was detected. Without Wnt, the luciferase detected by the reporter system was low (less than 500 relative light units, Fig 9A). However, with the control plasmid the addition of Wnt increased the luciferase substantially (to approximately 3500 relative light units). The PPIs had little effect on the luciferase emission in the control plasmid cells where the AHR expression is non-existent. The AHR plasmid transfected cells all emitted much less luciferase than the control plasmid transfected cells (less than 1000 relative light units compared with 3500), indicating that the AHR has an inhibitory effect on Wnt signaling. Remarkably, the PPIs further reduced Wnt-mediated luciferase in the presence of the AHR. These results indicate that the PPIs decrease Wnt signaling in an AHR-dependent manner.

For additional confirmation that the PPIs are affecting Wnt signaling through the AHR in TED, the phosphorylation of GSK3β, an important event in the Wnt signaling pathway, was measured in TED orbital fibroblasts using Western blotting (Fig 9B). TED orbital fibroblasts were first transfected with control siRNA or *AHR* specific siRNA. Then the fibroblasts were treated daily with esomeprazole or lansoprazole for 4 days and 1 ng/mL TFGβ was added after the first day. At the conclusion of the experiment cell lysates were measured for phosphorylated and total GSK3β levels with Western blotting. Both esomeprazole and lansoprazole at the 25 μM dose decreased phospho-GSK3β levels compared with TGFβ alone (lanes 4 and 6 versus lane 2; Fig 9B). This decrease in phospho-GSK3β by the PPIs was also AHR dependent. The PPIs no longer lowered phospho-GSK3β expression in lanes 7–12 when *AHR* specific siRNA was used. These data demonstrate that the PPIs block the β-catenin/Wnt pathway in an AHR dependent manner, similar to the results seen with the AHR ligand FICZ (9).

## Discussion

Current therapies aimed at reducing the symptoms and the tissue remodeling in the orbit during TED are imperfect. Not all patients will respond well and some patients that initially respond to therapy may relapse [2]. Multiple surgeries may be required to mediate disfigurement and/or save vision in the severest of cases and many patients remain unhappy with the outcome of treatment once concluded [2]. Alternatives and/or novel therapies to be used concomitantly with the standard treatments have the potential to benefit patients and are being actively investigated [2,7,23,24]. PPIs may be ideal candidates for further investigation as TED therapies.

Our data demonstrate that esomeprazole and lansoprazole can decrease TGFβ-induced αSMA and calponin expression, two hallmarks of myofibroblasts (Fig 1 and Fig 2). Additionally, our data show that PPIs prevent TGFβ-induced collagen production from TED orbital fibroblasts (Fig 3 and Fig 4). Taken together, these data indicate that the PPIs are potentially useful in preventing fibrosis or scarring. PPIs have been shown to have beneficial effects in other models of fibrosis and diseases characterized by fibrosis. Rats treated with esomeprazole or lansoprazole had less liver fibrosis when treated with carbon tetrachloride and in an experimental model of rat non-alcoholic steatohepatitis [25, 26]. Esomeprazole reduced fibrosis in mouse and rat lung injury models [14, 27]. Prophylactic treatment of the rats with esomeprazole before induction of the lung injury also prevented gene expression changes associated with fibrosis [14].

All doses of the PPIs showed clear benefits in preventing TGFβ-mediated myofibroblast formation except for the 1 μM dose in one strain (Strain B). At this dose, lansoprazole increased αSMA compared with TGFβ alone and other treatments (Fig 1). Both esomeprazole and lansoprazole increased cell-associated collagen at the 1 μM dose compared with the other treatments (Fig 4). We did not test this dose of the PPIs to determine if it activated the AHR in our experiments. Perhaps this dose is too low to activate the AHR strongly enough to affect myofibroblast formation. The PPIs may also have additional non-AHR related effects at the 1 μM dose. It is also possible that these results are related to something unique to this strain, as all our samples are derived from different individuals. We do not know whether this patient was already taking PPIs before the samples were collected. This information will be collected from patients donating samples in the future.

The PPIs were also shown to reduce TGFβ-induced proliferation in TED orbital fibroblasts in both a BrdU assay of proliferation and in a wound healing assay (Fig 5 and Fig 6). PPIs have also been shown to inhibit other models of proliferation and migration. Esomeprazole inhibits serum-induced proliferation of lung fibroblasts and epithelial cells [14]. Omeprazole and lansoprazole inhibit polymorphonuclear neutrophil migration [28, 29] and omeprazole decreased migration of a breast cancer cell line that was shown to be dependent on AHR through *AHR* siRNA experiments [10, 30].

The PPIs are AHR activators in TED orbital fibroblasts and prevent TGFβ-mediated myofibroblast formation in an AHR-dependent fashion (Fig 7 and Fig 8). Our laboratory has also shown that ITE and FICZ prevent TED myofibroblast formation due to TGFβ in an AHR-dependent manner (9). There are a few pathways known to be important for TGFβ-driven myofibroblast formation, which include: Smad2/3, MAPK/Erk, Akt, and β-catenin/Wnt signaling [20, 31–33]. In our previous work, these pathways were all investigated to determine if AHR expression and/or FICZ and ITE affected them during TGFβ-induced myofibroblast formation. Only the β-catenin/Wnt pathway appeared to be affected by the AHR activators and AHR expression in TED orbital fibroblasts [9]. Therefore, in this current study we also determined that esomeprazole and lansoprazole are interrupting the TGFβ-induced myofibroblast formation through the AHR and β-catenin/Wnt signaling pathways.

**Fig 10:**
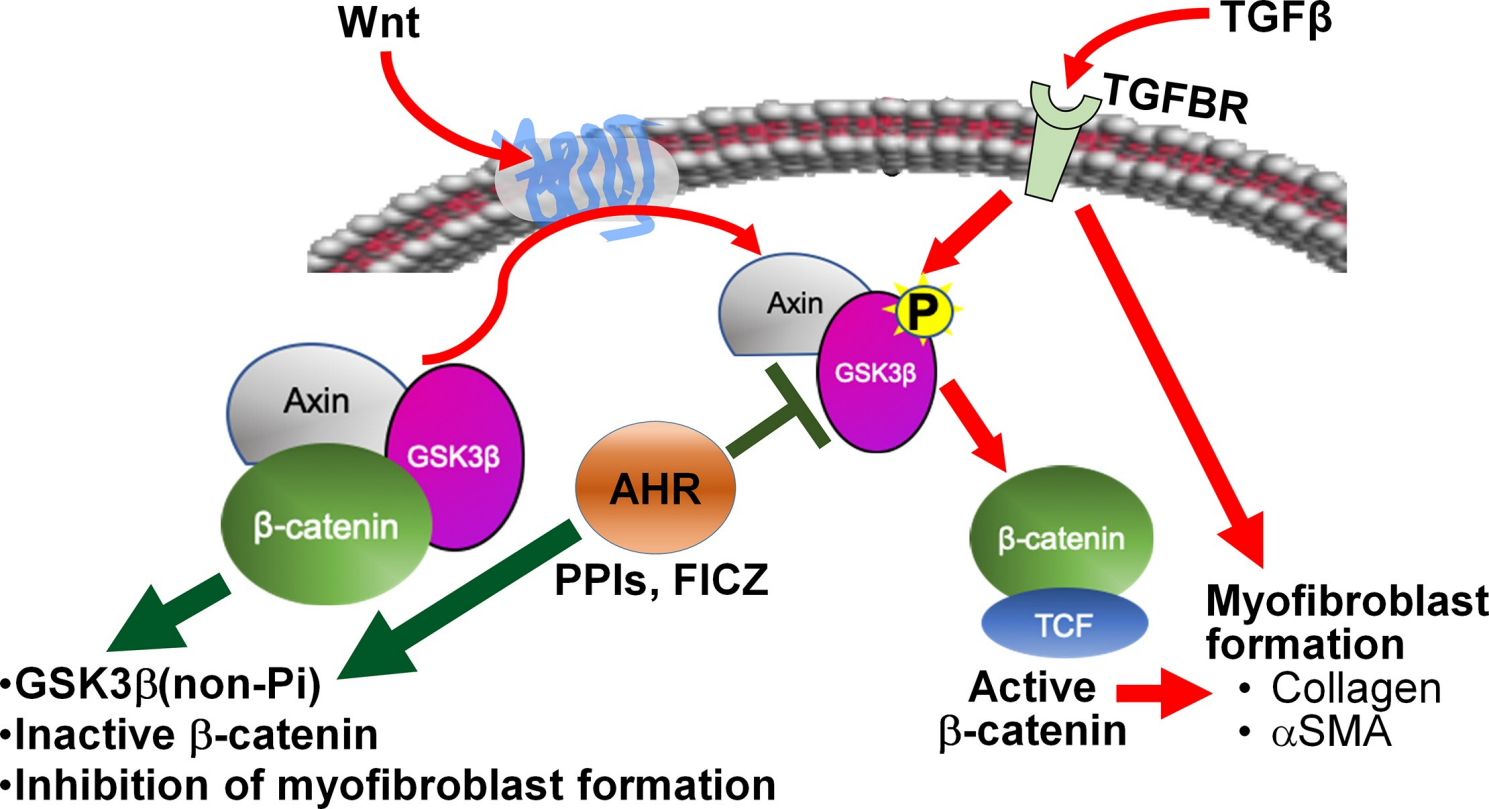
AHR prevents GSK3β phosphorylation to limit β-catenin/Wnt signaling and block myofibroblast formation. This is a simplified model of the potential interactions of TGFβ, AHR, and Wnt signaling pathways in TED, and how the PPIs may be signaling through AHR affecting Wnt signaling and preventing TGFβ-myofibroblast formation. TGFβ signaling induces myofibroblast formation through a few different pathways, one of which is the β-catenin/Wnt signaling pathway. TGFβ can directly phosphorylate GSK3β allowing β-catenin to enter the nucleus and promote the transcription of genes important for myofibroblast formation. Signaling through the AHR with PPIs or FICZ inhibits phosphorylation of GSK3β, thereby maintaining GSK3β and β-catenin in the destruction complex retained in the cytoplasm and blocking the transcription of β-catenin upregulated genes. Without β-catenin activated genes, myofibroblast formation is inhibited.

There appears to be crosstalk within the TGFβ, AHR, and β-catenin/Wnt signaling pathways, which is likely different in various tissues, disease states, and species and the importance of this interplay in the fibrotic process is beginning to be elucidated [31, 34–40]. TGFβ and AHR regulate the expression of the other in some species and cell lines [34–36]. In our investigation, we have discovered that the TGFβ, AHR, and β-catenin/Wnt signaling pathways appear to be working together in TED (Fig 10). The canonical pathway in TGFβ-mediated fibrosis is the Smad pathway; however, inhibition of this pathway does not completely block TGFβ-mediated fibrosis [31]. TGFβ can phosphorylate GSK3β alone, without the presence of Wnt [41]. Phosphorylation of GSK3β stabilizes β-catenin allowing the transcription of Wnt-responsive genes which include genes essential for myofibroblast formation [31, 42]. In TED orbital fibroblasts, signaling through the AHR inhibits the phosphorylation of GSK3β (Fig 9 and [9]), preventing the release of β-catenin from the destruction complex and favoring degradation of β-catenin over translocation to the nucleus and transcriptional activation. Another study also determined that the PPI pantoprazole inhibits β-catenin pathways in gastric cancer stem cells [43], suggesting that the ability of PPIs to block Wnt signaling may be important outside of TED as well.

Esomeprazole and lansoprazole had similar effects on myofibroblast formation; however, the efficacy of the two drugs was not directly compared in this study. In two areas, lansoprazole was a bit more effective than esomeprazole and these included preventing cell associated collagen production and wound closure (Fig 4 and Fig 6). For the prevention of collagen, lansoprazole had significant effects starting at the 25 μM dose; whereas, esomeprazole showed significant inhibition at the 50 μM dose (Fig 4). Lansoprazole also had a greater effect on preventing wound closure at earlier time points than esomeprazole as well as at lower doses (Fig 6). Both drugs seem to activate AHR and prevent β-catenin/Wnt signaling to a similar extent, although lansoprazole was able to increase *CYP1B1* expression to a greater extent at the 50 μM dose than esomeprazole (Fig 7A). A more direct comparison of esomeprazole and lansoprazole would likely provide insight as to which drug is a stronger candidate to investigate further for TED.

PPIs are among the most commonly prescribed drugs in the world [13]. After more than 25 years of use, PPIs have shown an excellent safety profile and although they are not completely innocuous they are well tolerated by most people [12, 44, 45]. Additionally PPIs are relatively cost effective treatments available both over the counter and with prescription [12, 44] Because of the need for new therapies for TED, the relative safety and low cost of PPIs and the potential benefit in reducing the numbers of myofibroblasts in TED, PPIs should be further investigated as drug candidates for TED patients. Retrospective studies of PPI use in TED patients should be performed to determine if there is an association between PPI use and prevention of TED development in Graves’ disease patients, as well as whether PPI use reduces the severity of TED. Future prospective studies on TED should also include criteria to investigate the use of PPIs in TED patients. These drugs may even be useful as prophylactic agents to prevent the development of TED in patients with Graves’ disease.

## Supporting information

S1 Fig Raw images of western blots preseneted in this manuscript. See the supplemental file S1 raw images for the uncropped Western blot images for Figs 1, 4, 7, 8 and 9.
(PDF)
