# Supplementary material for: Proton pump inhibitors attenuate myofibroblast formation associated with thyroid eye disease through the aryl hydrocarbon receptor
Source: PLoS One. 2019 Sep 19;14(9):e0222779. doi: 10.1371/journal.pone.0222779 (PMC6752849; doi:10.1371/journal.pone.0222779)
Supplement: S1 Fig — See the supplemental file S1 raw images for the uncropped Western blot images for Figs 1, 4, 7, 8 and 9. (PDF) [file pone.0222779.s001.pdf]

Strain A

Esomeprazole Fig 1A

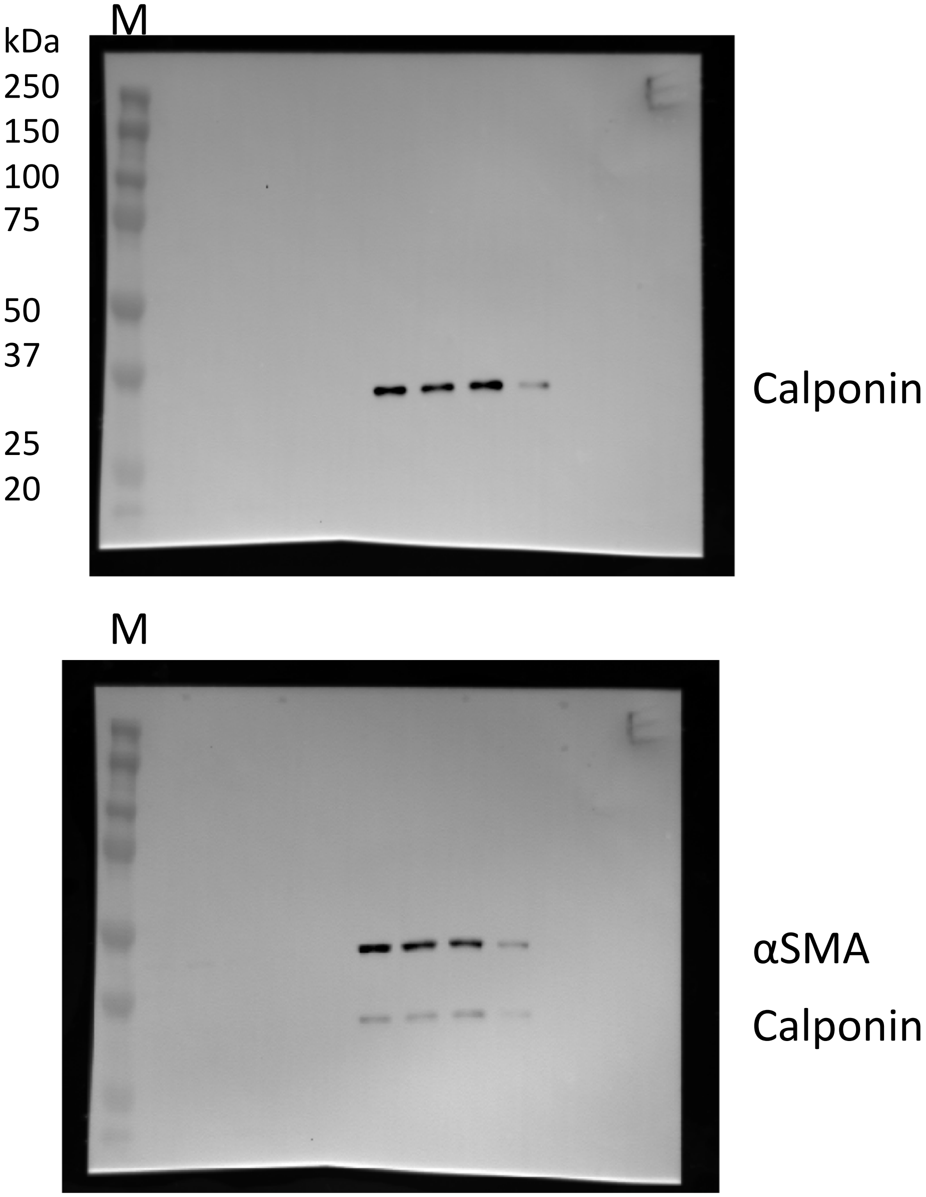

Chemiluminescent signals were captured using a VersaDoc Imaging System (Bio-Rad, Hercules, CA).

The molecular weight marker (M) is show on the left. All lanes are loaded as described in the figure. The blot probing order was Calponin, then αSMA, followed by tubulin.

Strain A
Esomeprazole Fig 1A

M

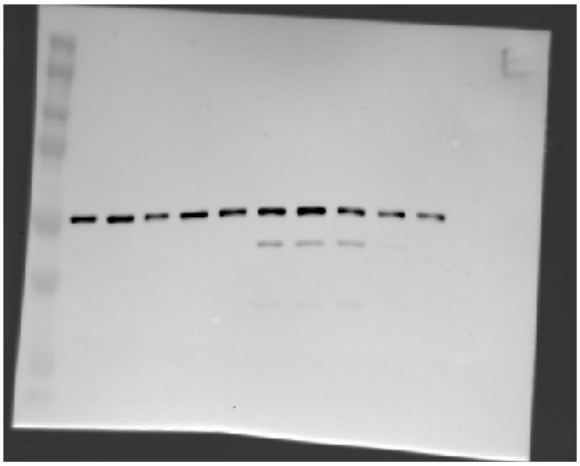

B-tubulin
αSMA
Calponin

Chemiluminescent signals were captured using a VersaDoc Imaging System (Bio-Rad, Hercules, CA).

The molecular weight marker (M) is show on the left. All lanes are loaded as described in the figure. The blot probing order was Calponin, then αSMA, followed by tubulin.

Strain B

Esomeprazole Fig 1A

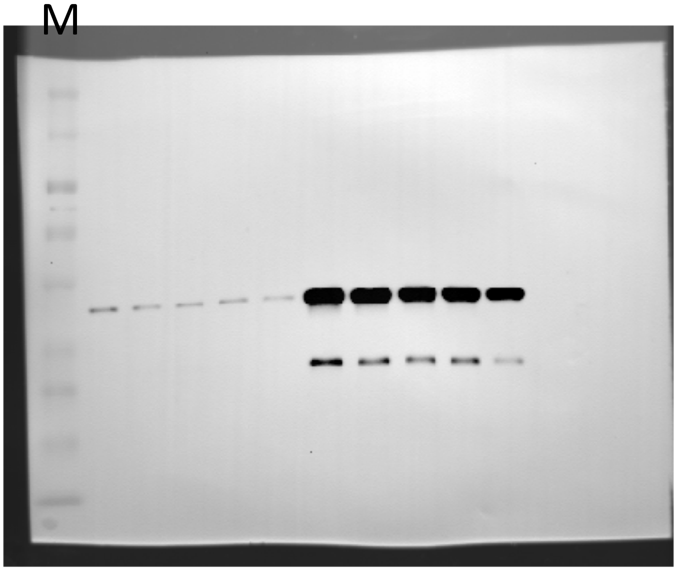

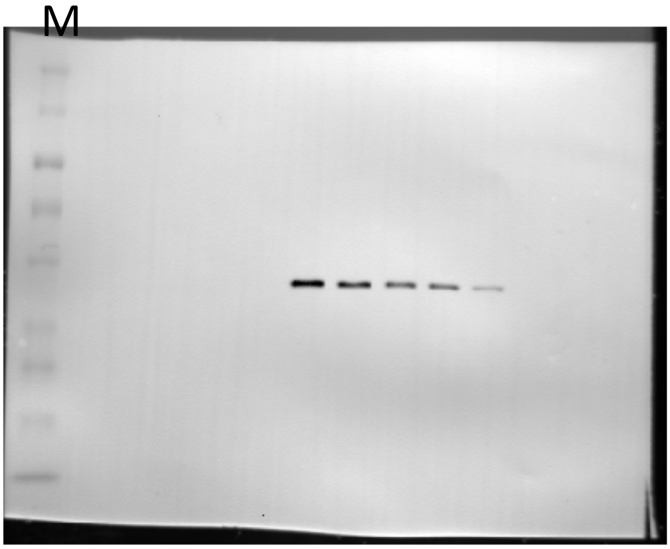

Chemiluminescent signals were captured using a VersaDoc Imaging System (Bio-Rad, Hercules, CA).

The molecular weight marker (M) is show on the left. All lanes are loaded as described in the figure. The blot probing order was Calponin, then αSMA, followed by tubulin.

Strain B
Esomeprazole Fig 1A

M

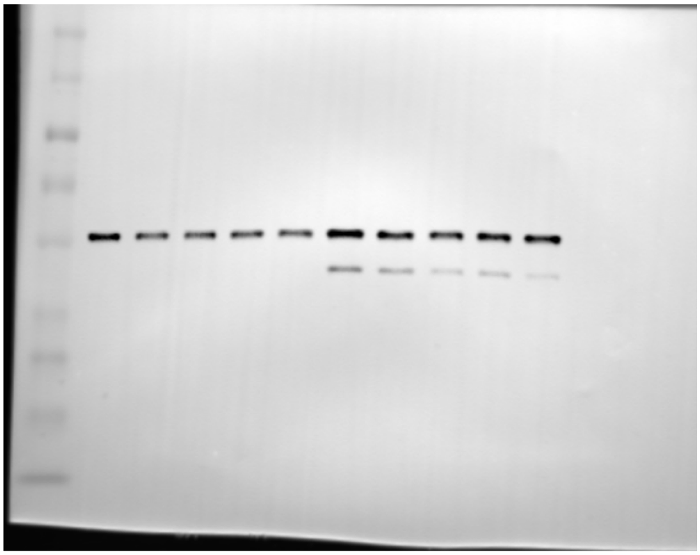

β-tubulin
αSMA

Chemiluminescent signals were captured using a VersaDoc Imaging System (Bio-Rad, Hercules, CA).

The molecular weight marker (M) is show on the left. All lanes are loaded as described in the figure. The blot probing order was Calponin, then αSMA, followed by tubulin.

Strain A

Lansoprazole Fig 1B

M

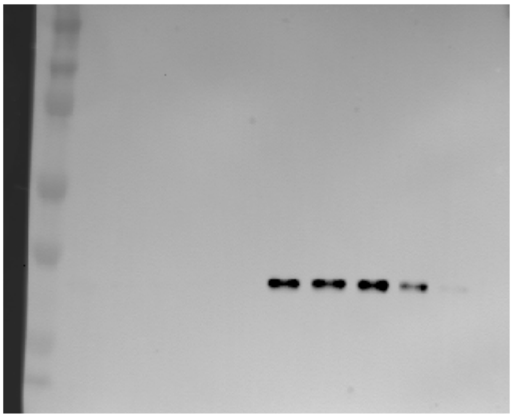

Calponin

M

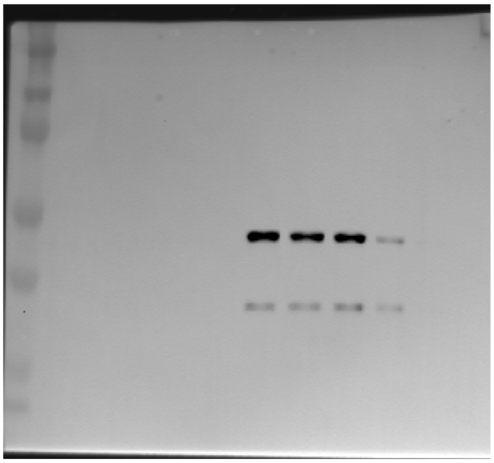

αSMA

Calponin

Chemiluminescent signals were captured using a VersaDoc Imaging System (Bio-Rad, Hercules, CA).

The molecular weight marker (M) is show on the left. All lanes are loaded as described in the figure. The blot probing order was Calponin, then αSMA, followed by tubulin.

Strain A

Lansoprazole Fig 1B

M

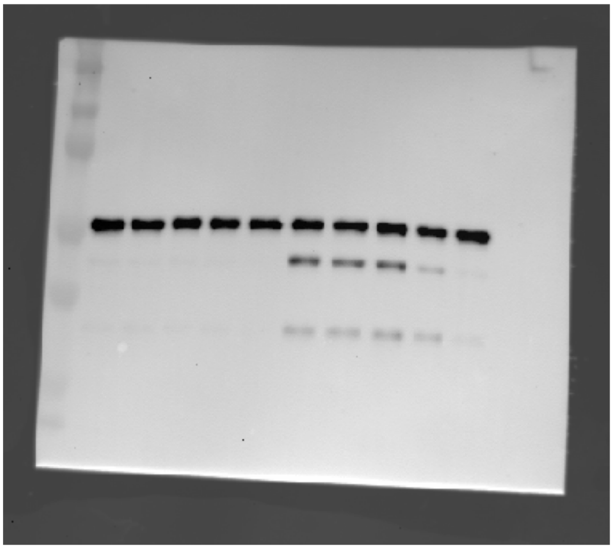

B-tubulin

αSMA

Calponin

Chemiluminescent signals were captured using a VersaDoc Imaging System (Bio-Rad, Hercules, CA).

The molecular weight marker (M) is show on the left. All lanes are loaded as described in the figure. The blot probing order was Calponin, then αSMA, followed by tubulin.

Strain B
Lansoprazole Fig 1B

M

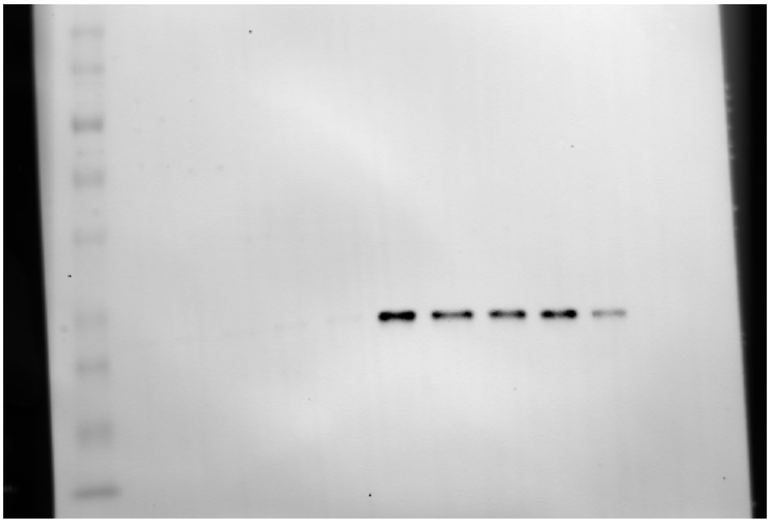

Calponin

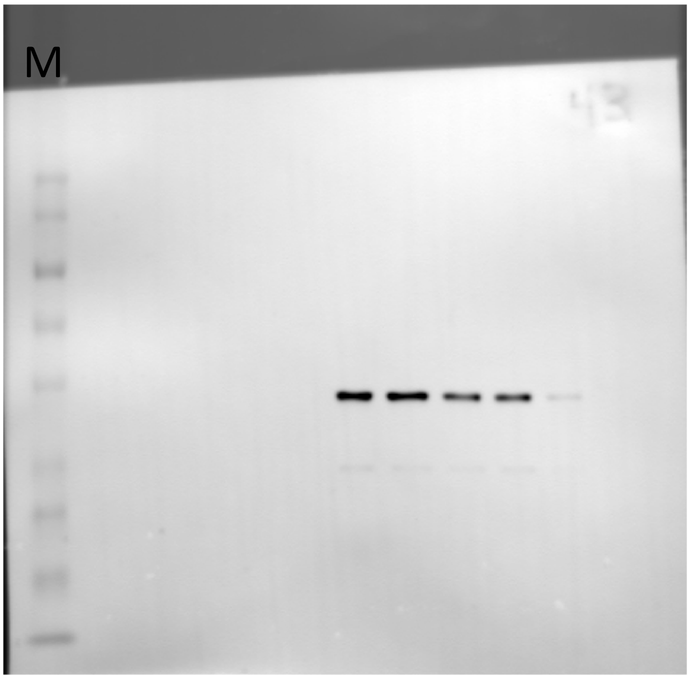

αSMA

Calponin

Strain B

Lansoprazole Fig 1B

M

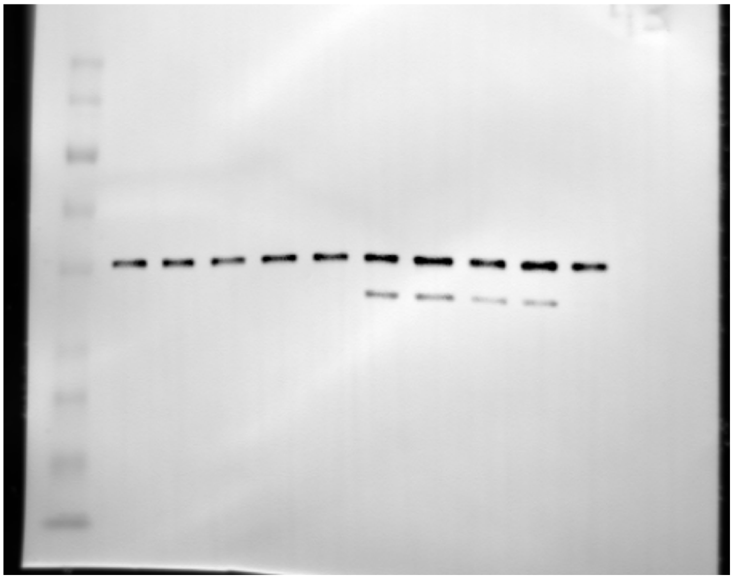

β-tubulin

αSMA

Chemiluminescent signals were captured using a VersaDoc Imaging System (Bio-Rad, Hercules, CA).

The molecular weight marker (M) is show on the left. All lanes are loaded as described in the figure. The blot probing order was Calponin, then αSMA, followed by tubulin.

Strain A
Esomeprazole Fig 4

M

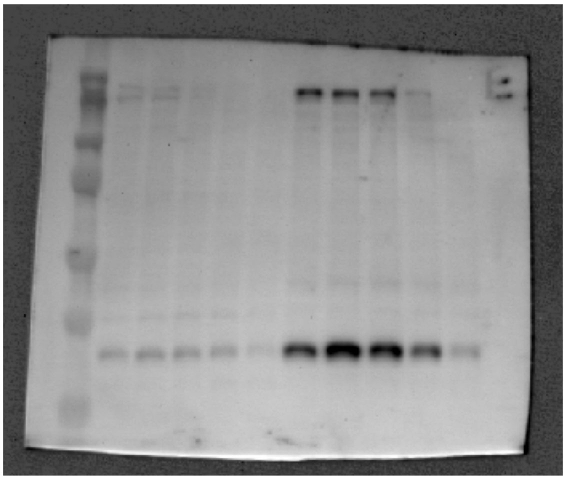

COL1A1

M

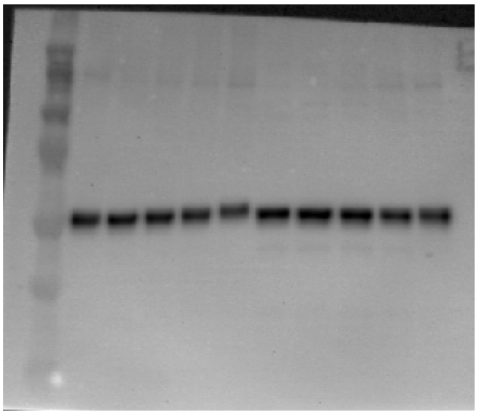

β-tubulin

Chemiluminescent signals were captured using a VersaDoc Imaging System (Bio-Rad, Hercules, CA).

The molecular weight marker (M) is show on the left. All lanes are loaded as described in the figure. The blot probing order was Calponin, then αSMA, tubulin and COL1A1.

Strain A

Lansoprazole Fig 4

M

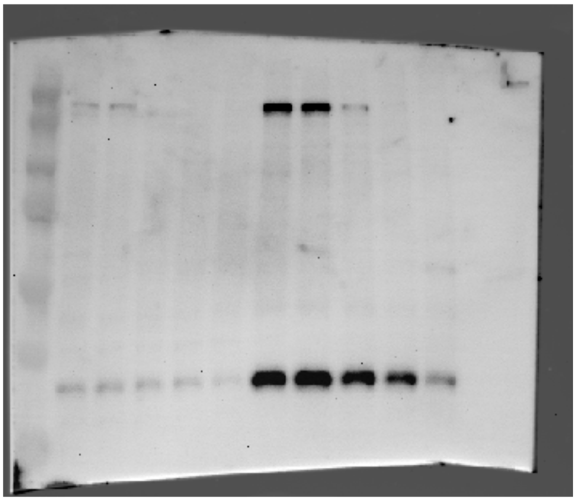

COL1A1

M

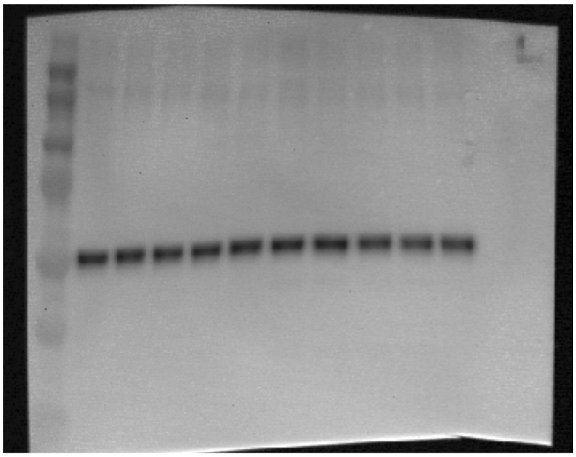

β-tubulin

Chemiluminescent signals were captured using a VersaDoc Imaging System (Bio-Rad, Hercules, CA).

The molecular weight marker (M) is show on the left. All lanes are loaded as described in the figure. The blot probing order was Calponin, then αSMA, tubulin and COL1A1.

Strain B
Esomeprazole Fig 4

M

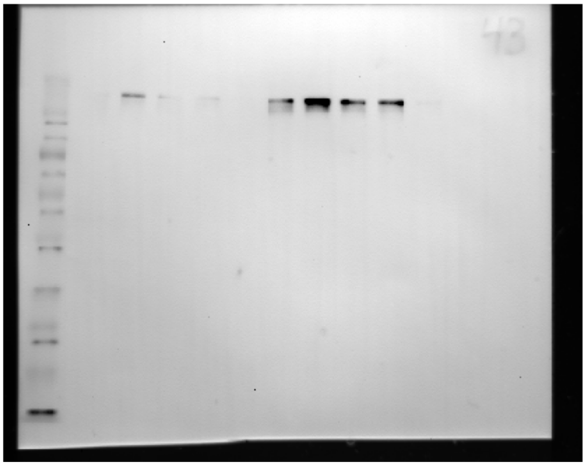

COL1A1

M

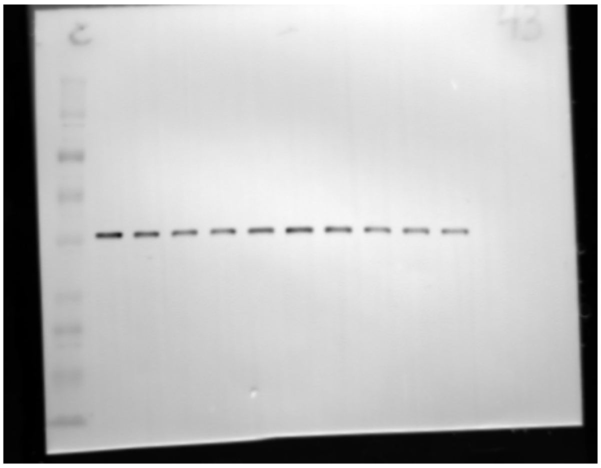

B-tubulin

Chemiluminescent signals were captured using a VersaDoc Imaging System (Bio-Rad, Hercules, CA).

The molecular weight marker (M) is show on the left. All lanes are loaded as described in the figure. The blot probing order was COL1A1 and then tubulin.

Strain B
Lansoprazole Fig 4

M

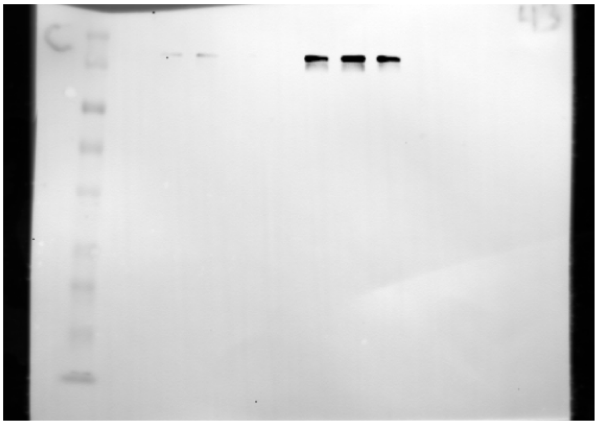

COL1A1

M

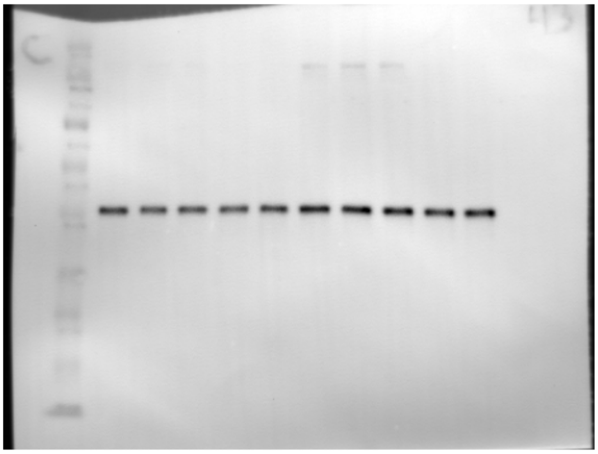

β-tubulin

Chemiluminescent signals were captured using a VersaDoc Imaging System (Bio-Rad, Hercules, CA).

The molecular weight marker (M) is show on the left. All lanes are loaded as described in the figure. The blot probing order was COL1A1 then tubulin.

Strain D
CYP1B1 Fig 7B

M

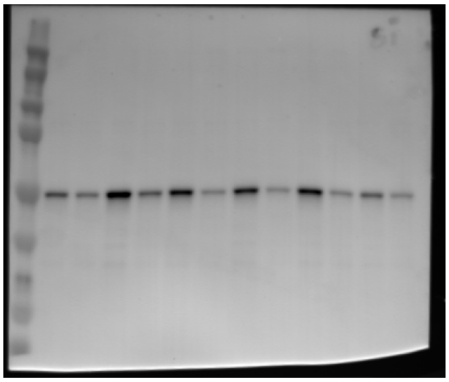

CYP1B1

M

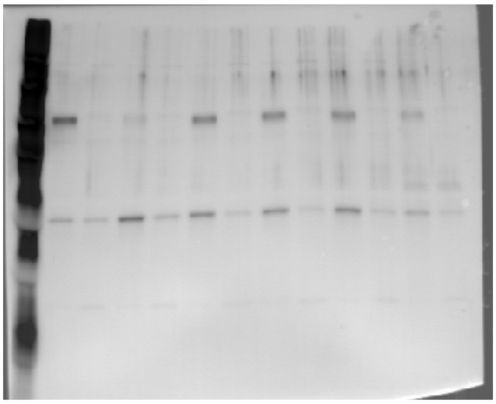

AHR

CYP1B1

Chemiluminescent signals were captured using a VersaDoc Imaging System (Bio-Rad, Hercules, CA).

The molecular weight marker (M) is show on the left. All lanes are loaded as described in the figure. The blot probing order was CYP1B1, AHR and then tubulin.

Strain D
CYP1B1 Fig 7B

M

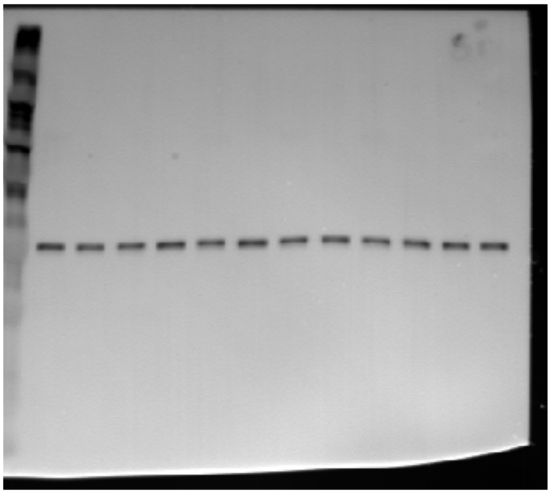

β-tubulin

Chemiluminescent signals were captured using a VersaDoc Imaging System (Bio-Rad, Hercules, CA).

The molecular weight marker (M) is show on the left. All lanes are loaded as described in the figure. The blot probing order was CYP1B1, AHR and then tubulin.

Strain E
CYP1B1 Fig 7B

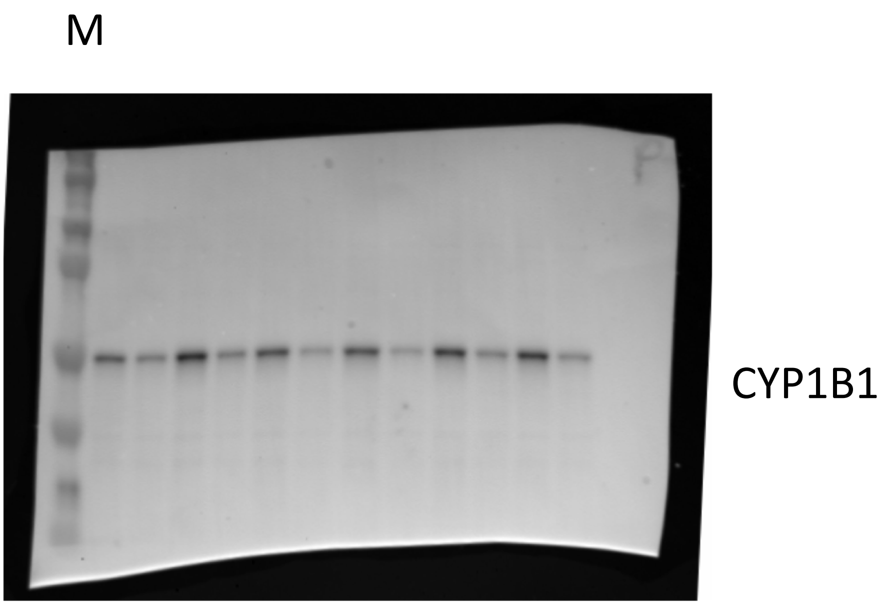

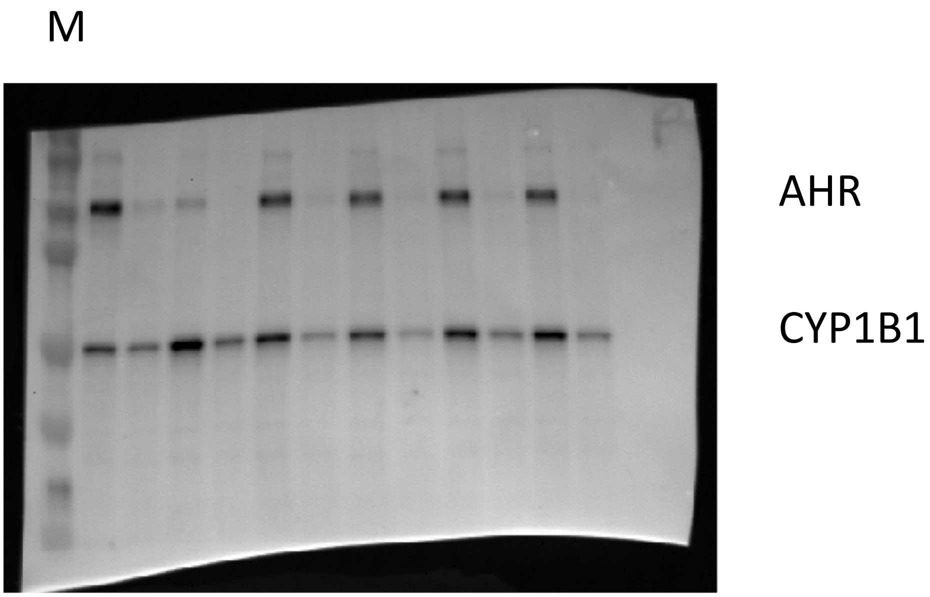

Chemiluminescent signals were captured using a VersaDoc Imaging System (Bio-Rad, Hercules, CA).

The molecular weight marker (M) is show on the left. All lanes are loaded as described in the figure. The blot probing order was CYP1B1, AHR and then tubulin.

Strain E
CYP1B1 Fig 7B

M

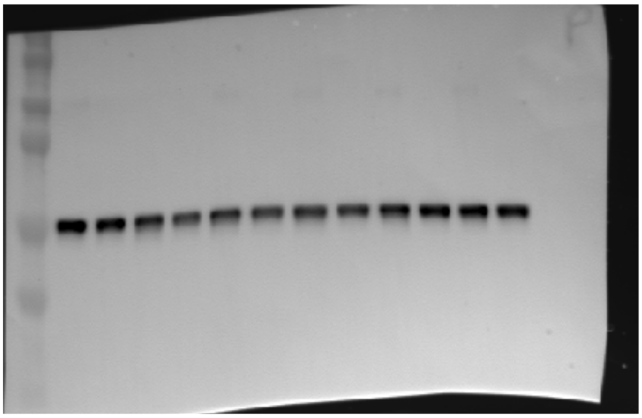

β-tubulin

Chemiluminescent signals were captured using a VersaDoc Imaging System (Bio-Rad, Hercules, CA).

The molecular weight marker (M) is show on the left. All lanes are loaded as described in the figure. The blot probing order was CYP1B1, AHR and then tubulin.

Strain A
AHR Dependence- Esomeprazole Fig 8

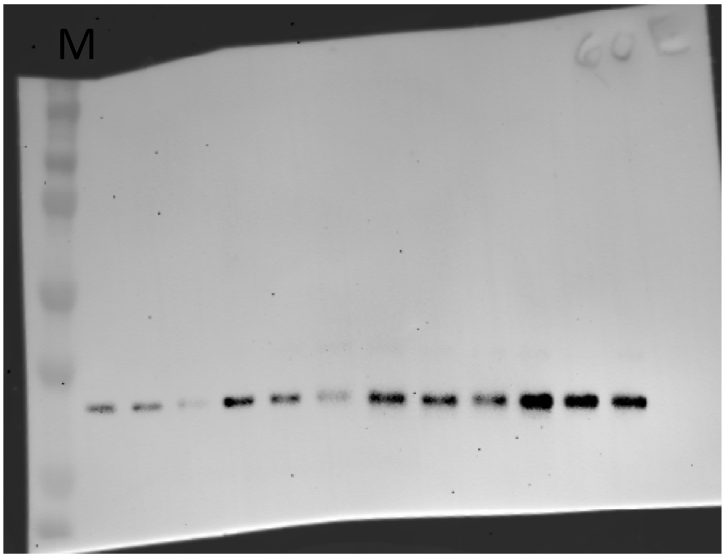

Calponin

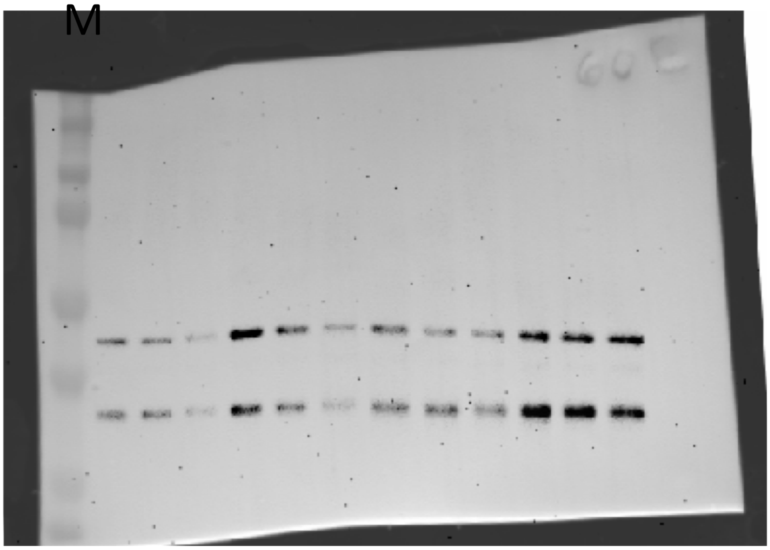

αSMA

Calponin

Chemiluminescent signals were captured using a VersaDoc Imaging System (Bio-Rad, Hercules, CA).

The molecular weight marker (M) is show on the left. All lanes are loaded as described in the figure. The blot probing order was Calponin, then αSMA, followed by tubulin.

Strain A
AHR Dependence- Esomeprazole Fig 8

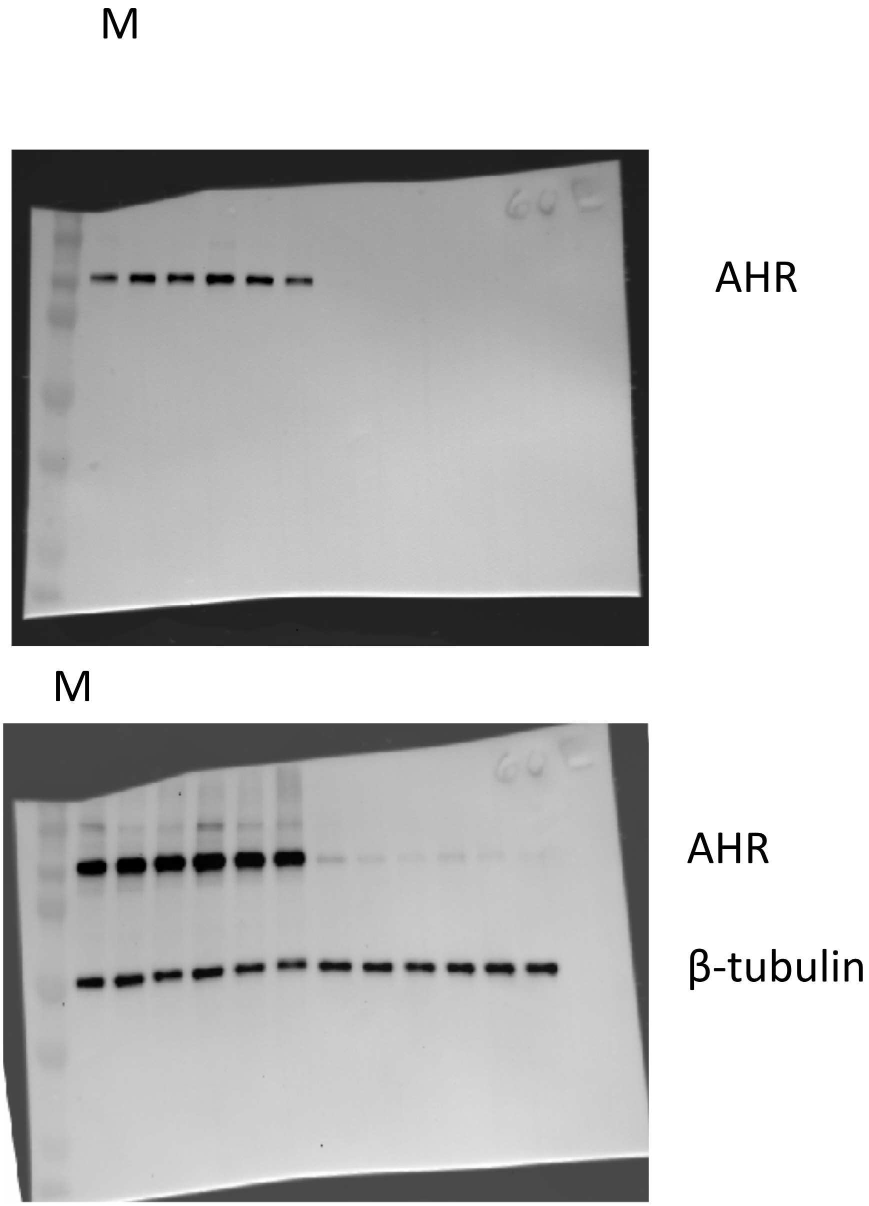

Chemiluminescent signals were captured using a VersaDoc Imaging System (Bio-Rad, Hercules, CA).

The molecular weight marker (M) is show on the left. All lanes are loaded as described in the figure. The blot probing order was AHR, followed by tubulin.

Strain A

AHR Dependence- Lansoprazole Fig 8

M

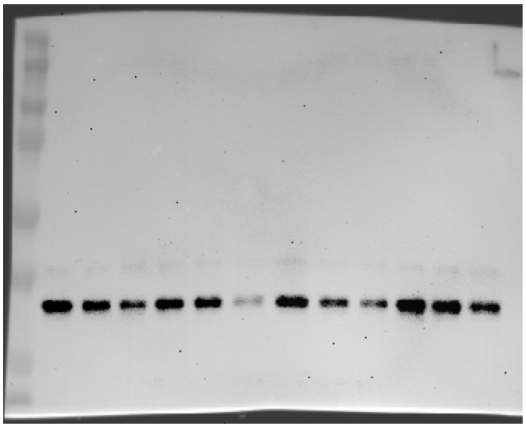

Calponin

M

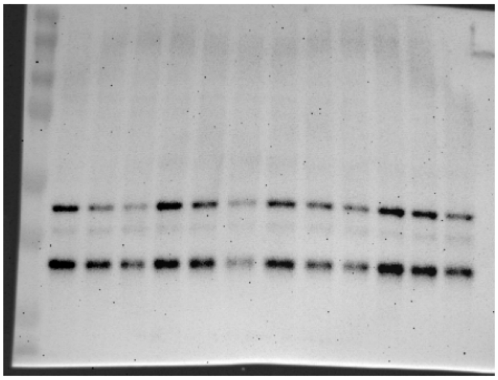

αSMA

Calponin

Chemiluminescent signals were captured using a VersaDoc Imaging System (Bio-Rad, Hercules, CA).

The molecular weight marker (M) is show on the left. All lanes are loaded as described in the figure. The blot probing order was Calponin, then αSMA, followed by tubulin.

Strain A
AHR Dependence- Lansoprazole Fig 8

M

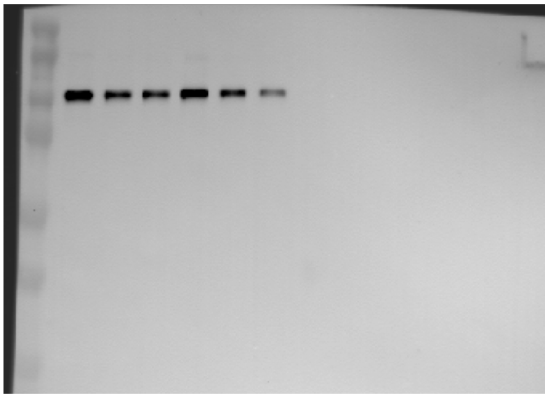

AHR

M

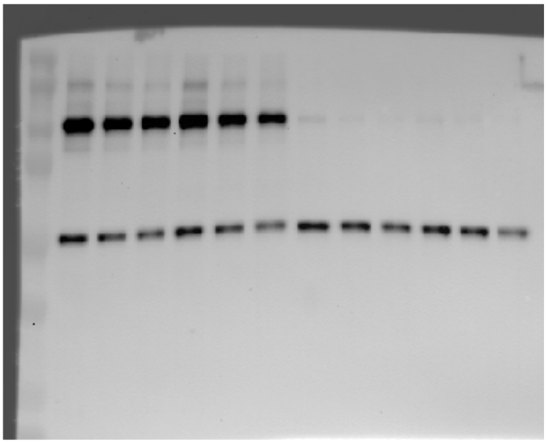

AHR

β-tubulin

Chemiluminescent signals were captured using a VersaDoc Imaging System (Bio-Rad, Hercules, CA).

The molecular weight marker (M) is show on the left. All lanes are loaded as described in the figure. The blot probing order was AHR, followed by tubulin.

Strain F

AHR Dependence- Esomeprazole Fig 8

M

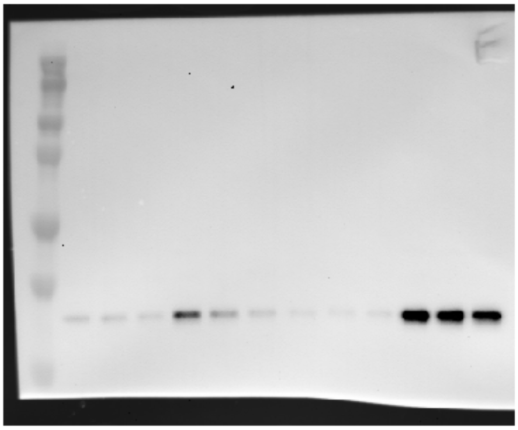

Calponin

M

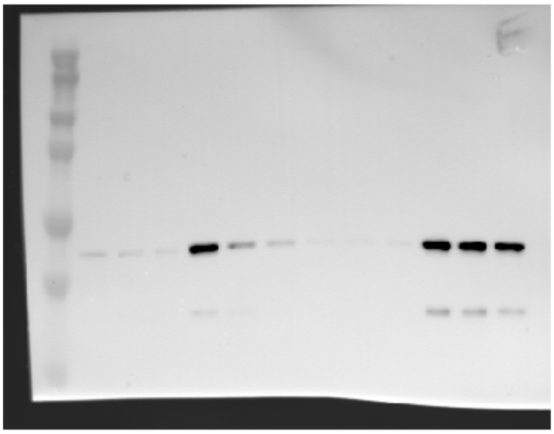

αSMA

Calponin

Chemiluminescent signals were captured using a VersaDoc Imaging System (Bio-Rad, Hercules, CA).

The molecular weight marker (M) is show on the left. All lanes are loaded as described in the figure. The blot probing order was Calponin, then αSMA, then AHR followed by tubulin.

Strain F

AHR Dependence- Esomeprazole Fig 8

M

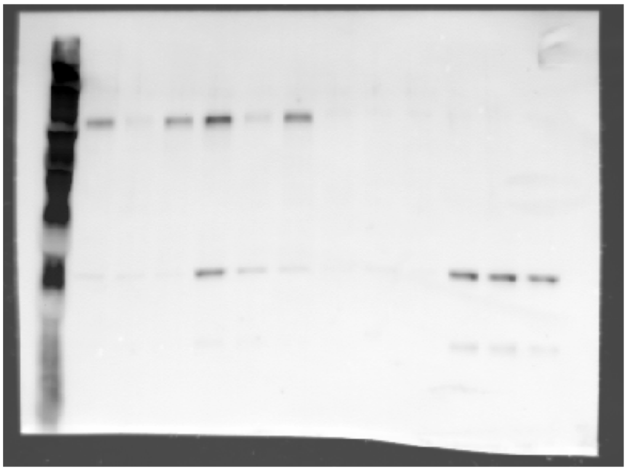

AHR

M

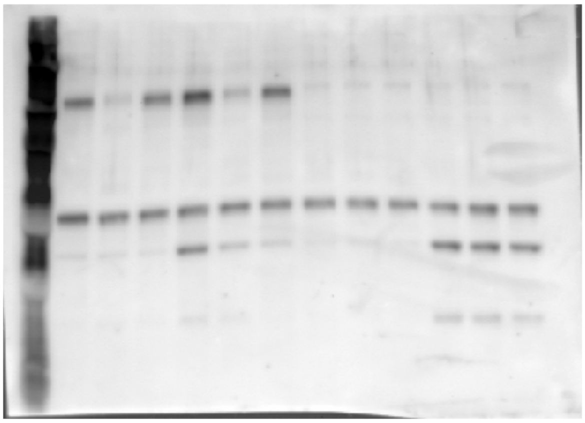

AHR

β-tubulin

αSMA

Calponin

Chemiluminescent signals were captured using a VersaDoc Imaging System (Bio-Rad, Hercules, CA).

The molecular weight marker (M) is show on the left. All lanes are loaded as described in the figure. The blot probing order was Calponin, then αSMA, then AHR followed by tubulin.

Strain F

AHR Dependence- Lansoprazole Fig 8

M

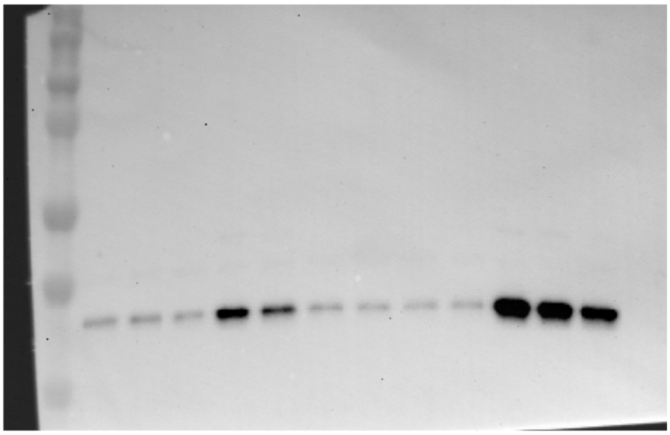

Calponin

M

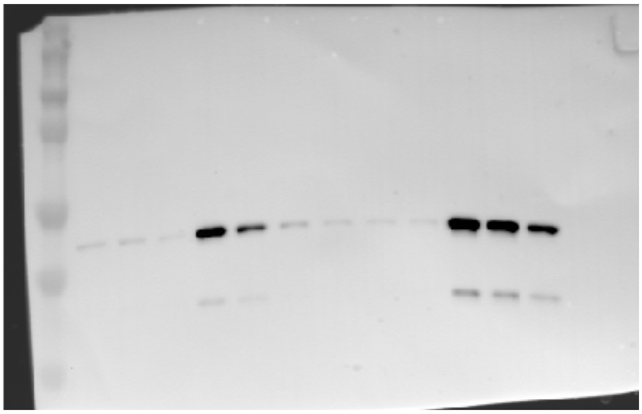

αSMA

Calponin

Chemiluminescent signals were captured using a VersaDoc Imaging System (Bio-Rad, Hercules, CA).

The molecular weight marker (M) is show on the left. All lanes are loaded as described in the figure. The blot probing order was Calponin, then αSMA, then AHR followed by tubulin.

Strain F
AHR Dependence- Lansoprazole Fig 8

M

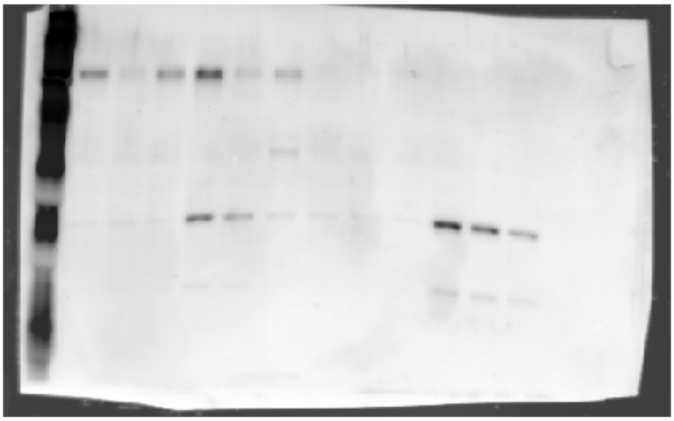

AHR

αSMA

Calponin

M

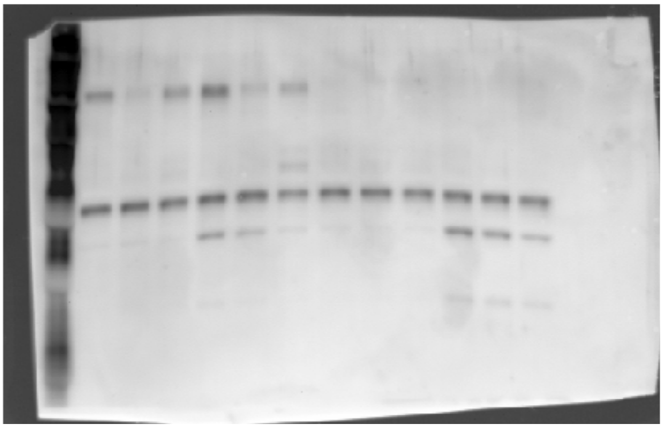

AHR

AHR
β-tubulin
αSMA
Calponin

Chemiluminescent signals were captured using a VersaDoc Imaging System (Bio-Rad, Hercules, CA).

The molecular weight marker (M) is show on the left. All lanes are loaded as described in the figure. The blot probing order was Calponin, then αSMA, then AHR followed by tubulin.

Strain A

GSK3B Fig 9B

M

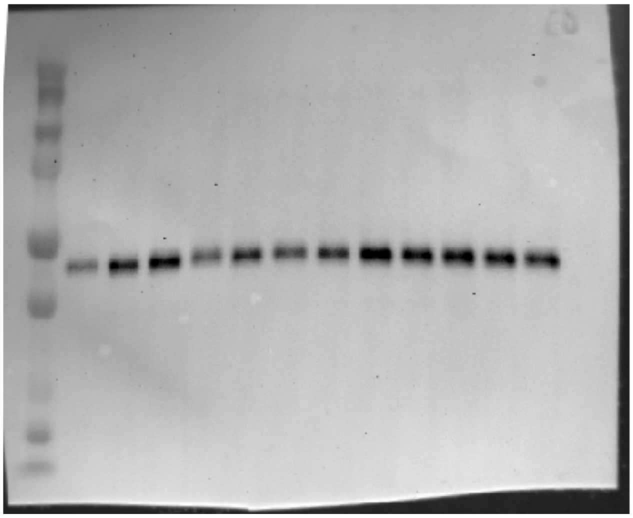

P-GSK3β

M

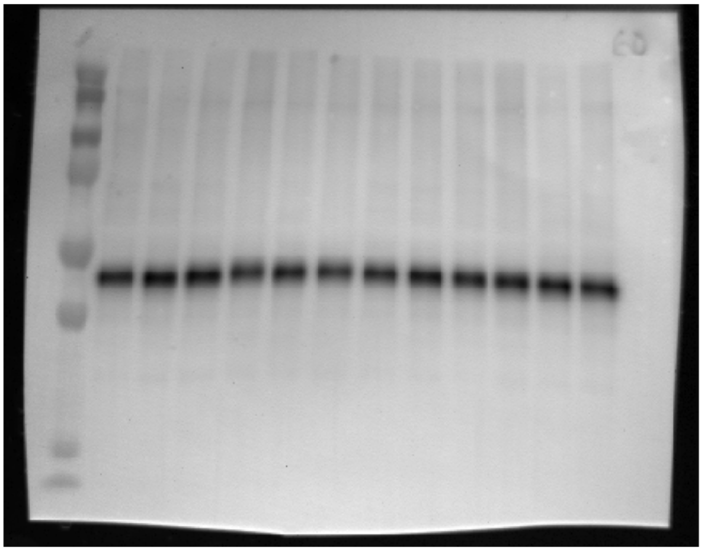

Total GSK3β

Chemiluminescent signals were captured using a VersaDoc Imaging System (Bio-Rad, Hercules, CA).

The molecular weight marker (M) is show on the left. All lanes are loaded as described in the figure. The blot probing order was phospho-GSK3β followed by total GSK3β and then tubulin.

Strain A

GSK3B Fig 9B

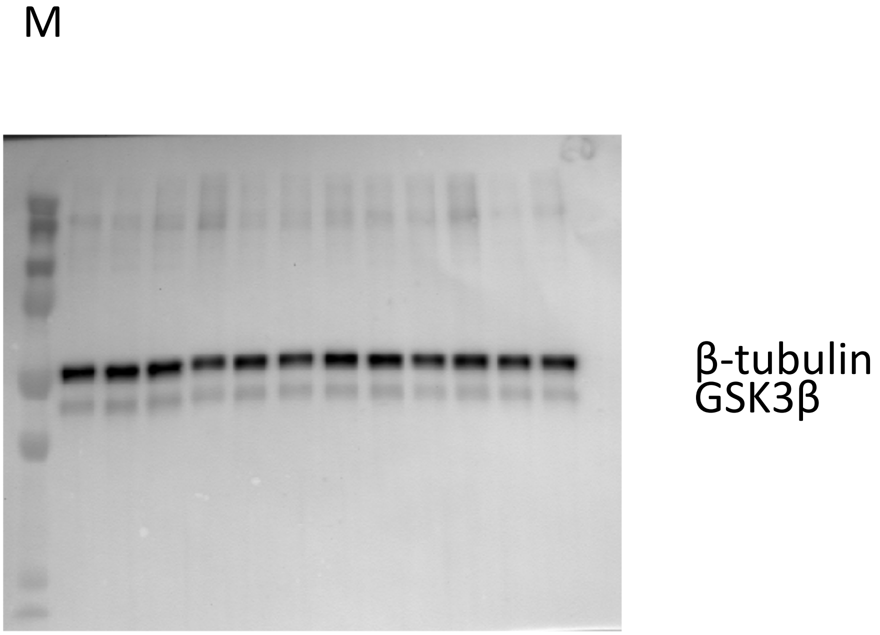

Chemiluminescent signals were captured using a VersaDoc Imaging System (Bio-Rad, Hercules, CA).

The molecular weight marker (M) is show on the left. All lanes are loaded as described in the figure. The blot probing order was phospho-GSK3β followed by total GSK3β and then tubulin.
